# The two-component system CpxAR controls biofilm formation by directly regulating the T3SS needle tip protein EseB in *Edwardsiella piscicida*

**DOI:** 10.1128/aem.02264-24

**Published:** 2025-05-29

**Authors:** Shu Ya Zhang, Shan Shan Sun, Lu Yi Liu, Thusyakaanth Sivaranjan, Pin Nie, Hai Xia Xie

**Affiliations:** 1 State Key Laboratory of Breeding Biotechnology and Sustainable Aquaculture, Institute of Hydrobiology, Chinese Academy of Sciences 543760, Wuhan, China; 2 College of Advanced Agricultural Sciences, University of Chinese Academy of Sciences 617064 , Beijing, China; 3 Wuhan Academy of Agricultural Sciences 726416, Wuhan, China; INRS Armand-Frappier Sante Biotechnologie Research Centre, Laval, Quebec, Canada

**Keywords:** CpxAR, T3SS needle tip, biofilm, *Edwardsiella piscicida*

## Abstract

The type III secretion system (T3SS) translocon protein EseB (needle tip protein) forms filamentous appendages on the surface of *Edwardsiella piscicida* to facilitate autoaggregation and biofilm formation. By contrast, another T3SS translocon protein EseC inhibits biofilm formation by sequestering EseC’s chaperone EseE, which also functions as a positive regulator of the *escC–eseE* operon, in which EseB is encoded. The two-component system (TCS) EsrAB and the regulator EsrC tightly and positively regulate the T3SS in *E. piscicida*. The TCS CpxAR provides an adaptive response to external environmental changes. In this study, we have shown that disruption of the histidine kinase CpxA (sensor) instead of CpxR (response regulator) significantly reduces biofilm formation in *E. piscicida*. CpxR is negatively regulated by CpxA, and significant amounts of CpxR accumulate in *E. piscicida* in the absence of CpxA. CpxR, together with EsrB and EsrC, directly binds the promoter of the *cpxR–cpxA* operon to promote CpxR transcription and expression. The elevated phosphorylated CpxR (CpxR–*P*) binds to the promoter of the *escC–eseE* operon to repress *eseB* transcription and expression, while EseE, EsrB, and EsrC bind directly to the same promoter to promote EseB transcription and expression. *E. piscicida* is an enteric pathogen that senses microbiota-derived indole in the gut lumen. EseB filament-mediated biofilm formation in *E. piscicida* is inversely proportional to exogenous indole. Together, CpxR inhibits while EsrB, EsrC, and EseE stimulate transcription and expression of the *escC–eseE* operon, thereby coordinately controlling EseB filament-mediated biofilm formation in *E. piscicida* in response to environmental stimuli.

**IMPORTANCE:**

*Edwardsiella piscicida* is primarily an enteric pathogen of fish and can form a biofilm to resist the lethal effects of host or antimicrobial agents. The assembly of filamentous appendages on the bacterial surface, mediated by the type III secretion system (T3SS) needle tip protein EseB, promotes bacterial-bacterial interactions and biofilm formation when *E. piscicida* is cultured in Dulbecco’s modified Eagle’s medium (DMEM). In this study, we have shown that the histidine kinase CpxA regulates biofilm formation in *E. piscicida* by negatively regulating its response regulator CpxR. Binding to the promoter of the *escC–eseE* operon, CpxR negatively regulates, whereas EsrB, EsrC, and EseE positively regulate the *escC–eseE* operon, of which EseB is encoded, coordinately regulating biofilm formation in *E. piscicida*.

## INTRODUCTION

*Edwardsiella piscicida* PPD130/91, formerly known as *Edwardsiella tarda* PPD130/91, is a Gram-negative enteric bacterium that infects more than 20 species of piscine hosts and is also an emerging agent of human infection (1, 2).

Culturing *E. piscicida* in Dulbecco’s modified Eagle’s medium (DMEM) switches on its type III secretion system (T3SS), and the substantial increase in expression and secretion of the T3SS needle tip protein EseB promotes EseB filament assembly on its surface. The EseB filament-mediated bacterial cell-cell interaction induces biofilm formation in *E. piscicida* (3, 4). In addition to EseB, the expression of the other two translocon proteins, EseC and EseD, is also increased when cultured in DMEM (5, 6). EseC and EseD can form pores on the host membrane through which T3SS effectors are translocated into the host. EseE is the chaperone of EseC, and it binds to EseC and facilitates EseC secretion (7). Upon EseC secretion, the released EseE (free EseE) positively regulates the *escC–eseE* operon, resulting in increased transcription and expression of EseB, thereby promoting EseB-mediated biofilm formation (4).

Biofilm formation is the process by which bacterial cells attach to a biotic or abiotic surface and become embedded in a matrix, transforming the properties of the bacteria from a free-living planktonic state to a multicellular, community-based state (8, 9). Biofilm formation can protect bacteria not only from conventional antimicrobial agents but also from host-derived cytokines due to the impermeable nature of the extracellular matrix in biofilm. Biofilm is also the source of recurrent infection (10, 11). Biofilm formation in some bacteria is regulated by two-component systems (TCSs) that sense changes in environmental stimuli and the bacterial envelope, helping bacteria to adapt to fluctuating environments (12–19). The TCS GacSA and TctDE in *Pseudomonas aeruginosa* (13, 14), BbeR-BbeS in *Burkholderia pseudomallei* (15), CpxAR in *Salmonella* Enteritidis, avian pathogenic *Escherichia coli* and *Yersinia pseudotuberculosis* (16–18), and EsrAB in *E. piscicida* (19) are involved in the regulation of biofilm formation.

The TCS usually consists of a sensor histidine kinase (HK) and a cytoplasmic response regulator (RR). The histidine kinase CpxA (sensor) is located in the inner membrane and the response regulator CpxR in the cytoplasm of the bacterial cell, and CpxP, a co-regulatory protein, is located in the periplasm of the bacterial cell (20). Upon activation, CpxA is autophosphorylated, and the phosphorylated CpxA functions as a kinase, transferring its phosphoryl group to the conserved aspartate in CpxR. The phosphorylated CpxR (CpxR–*P*) regulates the expression of target genes associated with periplasmic protein folding, degradation factors, antibiotic resistance, peptidoglycan metabolic enzymes, inner membrane proteins and regulators, etc. (20–24). CpxA also acts as a phosphatase, removing phosphate residues from CpxR–*P* (17). Deletion of *cpxA* results in a specific kinase deficiency and reduced phosphatase activity, and in such a scenario, CpxR could be phosphorylated through the phosphotransacetylase (Pta)-acetate kinase (AckA) pathway, as reported in *Y. pseudotuberculosis* (25). Acetylphosphate is a high-energy compound consisting of an acetyl and a phosphate group produced by the AckA-Pta pathway, and by transferring the phosphoryl group from acetylphosphate to CpxR, CpxR could be phosphorylated (21, 23). The increased CpxR–*P* represses the Ysc-Yop T3SS of *Y. Pseudotuberculosis* and inhibits the expression of *Salmonella* Pathogenicity Island 1 (SPI-1) genes by affecting the stability of HilD (25, 26). In *Yersinia*, CpxAR suppresses biofilm formation by limiting the production of exopolysaccharide (EPS), the core component of the biofilm extracellular matrix (18). In the uropathogenic *Proteus mirabilis*, CpxR positively regulates the expression of MR/P fimbriae and ZapABCD, and the production of fimbriae and ZapBCD-associated EPS facilitates biofilm formation (12).

The TCSs are interconnected to coordinately regulate virulence in some bacterial pathogens. The response regulator CpxR binds directly to the promoter of *ssrAB* to exert negative regulation in *Salmonella* (27). The TCS EsrAB, an ortholog of SsrAB in *S. enterica*, regulates the *E. piscicida* T3SS either directly or indirectly through regulation of the regulatory protein EsrC (28). Upon sensing environmental iron signals, the TCS response regulator BasR binds to the *esrB* promoter to modulate *E. piscicida* T3SS (29, 30).

Indole is an important intercellular signaling molecule in enteric pathogens (31). It is produced from L-tryptophan catalyzed by tryptophanase (TnaA) with pyridoxal 5′-phosphate (PLP) as a coenzyme or by the indole synthase AbiS (32, 33). *E. piscicida* can produce indole, and its concentration peaks at 35.5 µmol/L when the OD_600_ reaches ~1.9 in tryptic soy broth (TSB) (34). As an enteric pathogen, *E. piscicida* encounters microbiota-derived indole in the gut lumen. The *Salmonella* T3SS needle tip protein SipD binds to 5-hydroxyindole, and the translocon protein SipB could interact with both 3-indoleacetic acid and 5-hydroxyindole, resulting in T3SS deactivation or shutdown (35).

In this study, we have shown that in the absence of CpxA, an elevated level of CpxR represses transcription of the *escC–eseE* operon, which encodes EseB in *E. piscicida*. Meanwhile, EseE, together with EsrB and EsrC, binds to the promoter region of the *escC–eseE* operon and positively regulates EseB expression. The four regulators thus fine-tune EseB-filament-mediated biofilm formation in response to environmental cues such as indole or nutrients.

## RESULTS

### CpxA induces autoaggregation and biofilm formation in *E. piscicida*

**Fig 1:**
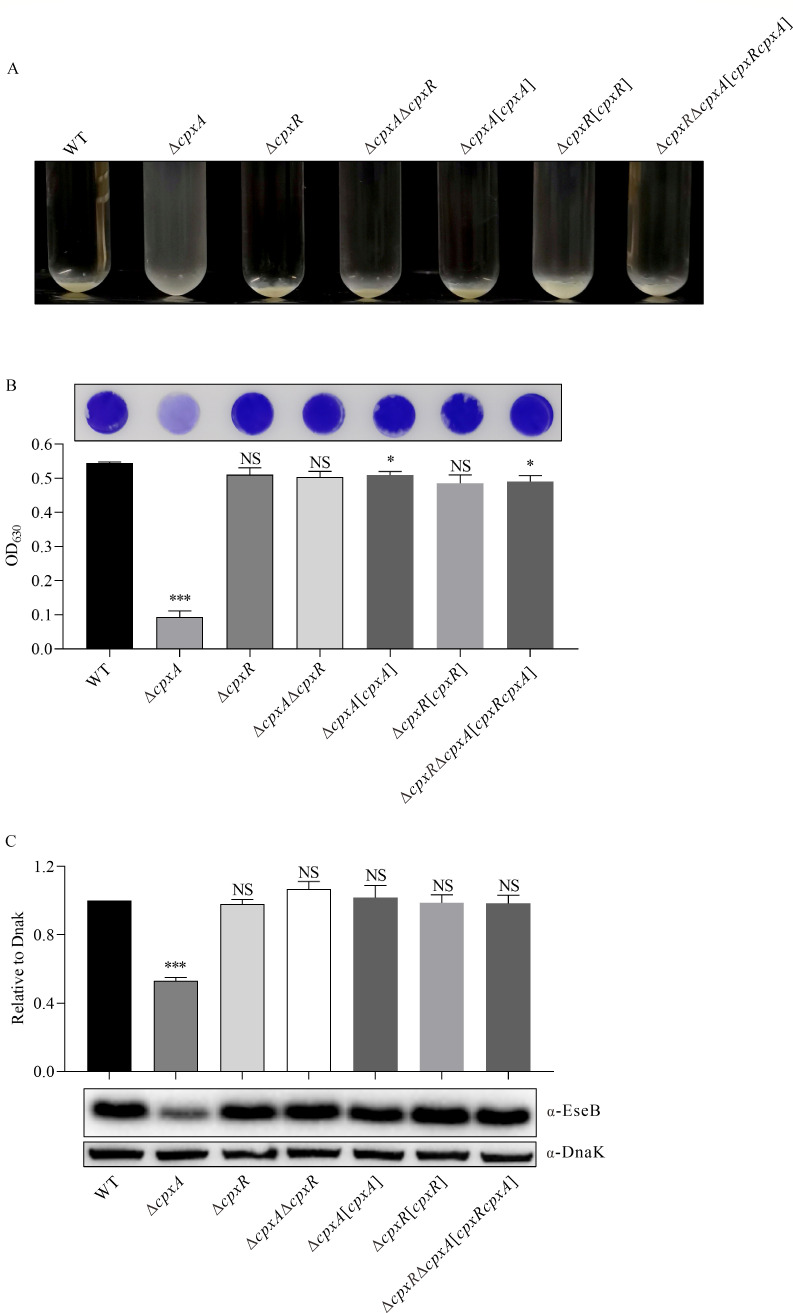
CpxA induces autoaggregation and biofilm formation in *E. piscicida*. (**A**) Autoaggregation of *E. piscicida* strains in DMEM at 25.0°C under a 5.0% CO_2_ atmosphere in a glass test tube at 24 hours post-subculture (hps). (**B**) Biofilm formed by *E. piscicida* strains in DMEM. *E. piscicida* strains were subcultured in DMEM in a 24-well plate horizontally embedded with coverslips, biofilms developed on the coverslips were fixed and stained with 0.2% crystal violet (left panel), and biofilm formation was evaluated by examining the OD_630_ of the dissolved crystal violet (right panel). ***P < 0.001; *P < 0.05; NS, not significant. (**C**) Immunoblotting of the steady-state protein levels of EseB in *E. piscicida* strains. Total bacterial proteins (TBPs) from equal amounts of *E. piscicida* strains were probed with rabbit anti-EseB and rabbit anti-DnaK antibodies. DnaK was used as a loading control (left panel). Protein levels of EseB from bacterial pellets were quantified by densitometry and normalized to DnaK. The graphs show the relative ratios of intracellular EseB, which are averages of the results of at least three independent experiments (right panel). ***P < 0.001; NS, not significant.

The TCS CpxAR responds to various stresses that affect bacterial envelope homeostasis (36). Assembly of EseB filaments on the surface of *E. piscicida* induces changes in the cell envelope and mediates autoaggregation and biofilm formation (3). Does CpxAR control autoaggregation and biofilm formation in *E. piscicida*? To investigate this, ∆*cpxA*, ∆*cpxR,* and ∆*cpxA*∆*cpxR* strains were constructed, and their complementary strains (∆*cpxA*[*cpxA*], ∆*cpxR*[*cpxR*], and ∆*cpxR*∆*cpxA*[*cpxRcpxA*]) were constructed by introducing a low-copy plasmid pWSK29 carrying a functional allele of *cpxA*, *cpxR,* or *cpxR–cpxA* together with the promoter upstream of *cpxR. E. piscicida* strains were subcultured into DMEM in glass tubes for 24 hours at static conditions. It was observed that each of the *E. piscicida* strains settled to the bottom of the glass tubes, causing their supernatants to become transparent, except for the ∆*cpxA* strain, which remained cloudy (Fig. 1A). This indicates that deletion of *cpxA* abolishes the autoaggregation of *E. piscicida* in DMEM. To assess the involvement of CpxA in biofilm formation, biofilms formed at the bottom of the cell culture wells were fixed and stained with crystal violet. It was observed that the WT, ∆*cpxR*, ∆*cpxA*∆*cpxR*, ∆*cpxA*[*cpxA*], ∆*cpxR*[*cpxR*], and ∆*cpxR*∆*cpxA*[*cpxRcpxA*] strains formed mature biofilm, whereas the ∆*cpxA* strain formed immature biofilm (Fig. 1B, left panel). Quantitative analysis showed that the biofilm formed by the ∆*cpxR*, ∆*cpxA*∆*cpxR*, ∆*cpxA*[*cpxA*], ∆*cpxR*[*cpxR*], and ∆*cpxR*∆*cpxA*[*cpxRcpxA*] strains was not significantly different from the WT strain, but the ∆*cpxA* strain formed significantly less developed biofilm (Fig. 1B, right panel). Furthermore, deletion of *cpxA* dramatically and significantly reduced the steady-state protein level of EseB (Fig. 1C). Taken together, deletion of *cpxA*, but not *cpxR*, impaired autoaggregation and biofilm formation in *E. piscicida*.

### CpxA promotes biofilm formation by upregulating EseB in *E. piscicida*

**Fig 2:**
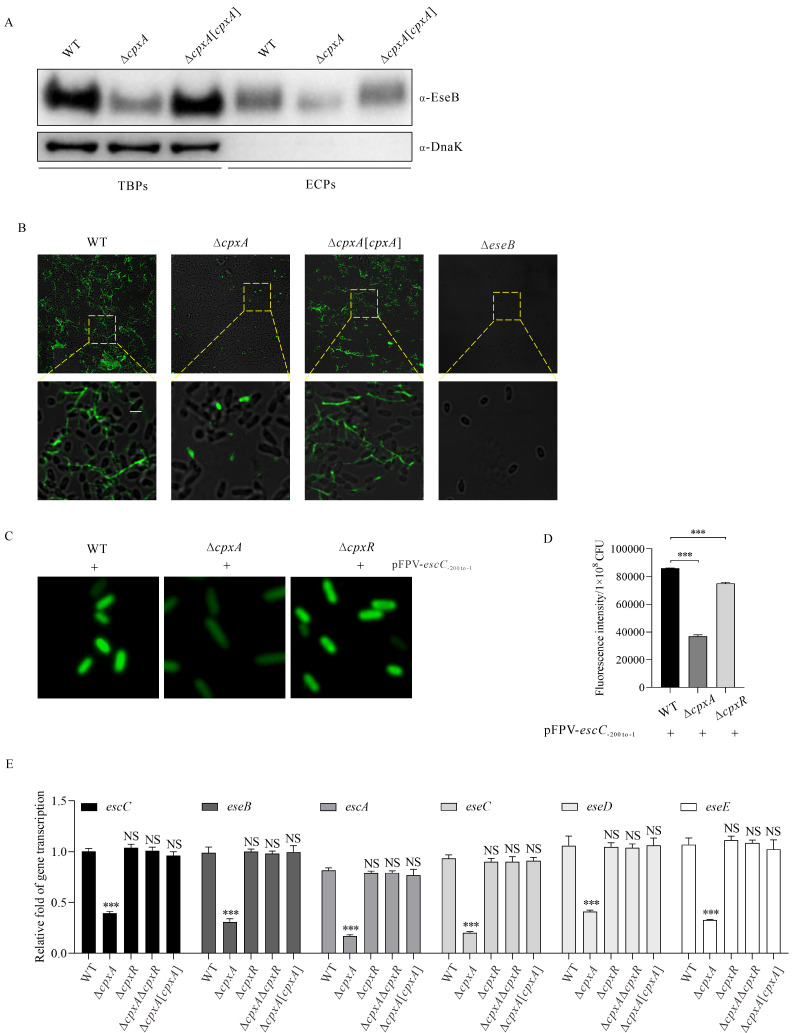
CpxA represses the expression and secretion of the T3SS translocon protein EseB. (**A**) Immunoblotting of EseB expression and secretion in *E. piscicida* strains. Total bacterial proteins (TBPs) and extracellular proteins (ECPs) from equal amounts of *E. piscicida* WT, ∆*cpxA*, and ∆*cpxA*[*cpxA*] strains were probed with rabbit anti-EseB and rabbit anti-DnaK antibodies. DnaK, a cytosolic chaperone, was used as the loading control. The immunoblotting data shown are representative of three independent experiments. (**B**) Immunofluorescence staining of *E. piscicida* strains with mouse anti-EseB antibody. *E. piscicida* WT, ∆*cpxA*, ∆*cpxA*[*cpxA*], and ∆*eseB* strains were subcultured in DMEM in a 24-well plate horizontally embedded with coverslips. The biofilm formed on the coverslips was immunofluorescently stained, and images were captured using a confocal laser scanning microscope. Scale bar, 5.0 µm, scale bar for magnification, 15.0 µm. (**C**) The immunofluorescence images of the *E. piscicida* WT*,* ∆*cpxA*, and ∆*cpxR* strains, each being introduced with pFPV-*escC*_-200 to -1_-*gfp*. At 24 hps in DMEM, the image for each strain was captured under a confocal microscope. Scale bar, 50.0 µm. (**D**) Fluorescence intensity of the GFP signal in *E. piscicida* WT, ∆*cpxA*, and ∆*cpxR* strains, each being introduced with pFPV25-*escC*_-200 to -1_-*gfp*. Fluorescence intensity indicates the protein level of GFP in each strain examined at 24 hps in DMEM. ***P < 0.001. (**E**) The mRNA levels of *escC*, *eseB*, *escA*, *eseC*, *eseD,* and *eseE* in the *escC–eseE* operon in *E. piscicida* strains were investigated by qRT-PCR. Multi-reference genes (*rpoB* and *gyrB*) were used to determine the relative transcript levels of each gene. Data are expressed as mean ± SD. One-way ANOVA in SPSS was used to calculate the *P* values as compared to the WT strain. ***P < 0.001; NS, not significant.

To investigate the underlying mechanism by which CpxA promotes biofilm formation, *E. piscicida* strains subcultured in DMEM were examined by immunoblotting. A reduced steady-state protein level of EseB was detected in the ∆*cpxA* strain compared to the WT strain, and the introduction of pWSK-*cpxR*_-762 to -1 _-*cpxA*-2HA into the ∆*cpxA* strain restored the protein level of EseB to that of the WT strain. Accordingly, significantly reduced EseB secretion was detected in the ∆*cpxA* strain (Fig. 2A). Notably, DnaK was not detected in extracellular proteins (ECPs), indicating that EseB in ECPs is not due to bacterial leakage.

To investigate how CpxA regulates the assembly of EseB filaments on the surface of *E. piscicida*, *E. piscicida* strains grown on coverslips at 24 hours post-subculture (hps) were subjected to immunofluorescence staining with mouse anti-EseB antibody and donkey anti-mouse IgG Alexa Fluor 488 antibody. Much shorter and far fewer EseB filaments were observed on the surface of the Δ*cpxA* strain than on the surface of the WT strain or the Δ*cpxA*[*cpxA*] strain. EseB filaments could not be detected on the surface of the Δ*eseB* strain (Fig. 2B). This suggests that deletion of *cpxA* strongly attenuates biofilm formation in *E. piscicida* by downregulating EseB secretion, thereby affecting the assembly of EseB filaments on the surface.

To understand why CpxA is required for the maximal levels of EseB, the promoter region of the *escC–eseE* operon was inserted before *gfp* in pFPV25 to give pFPV-*escC*_-200 to -1_-*gfp*. This plasmid was then introduced, respectively, into WT, ∆*cpxA,* and Δ*cpxR* strains, and their GFP signal intensities were compared. The weakest GFP signal was detected in the Δ*cpxA* strain, whereas a slightly reduced GFP signal was detected in the Δ*cpxR* strain as compared to the WT strain counterpart (Fig. 2C and D). To confirm this, the transcription of each gene in the *escC–eseE* operon was examined by quantitative real-time PCR (qRT-PCR). Significantly decreased transcription of *escC*, *eseB*, *escA*, *eseC*, *eseD*, and *eseE* was detected in the Δ*cpxA* strain compared to the WT strain, Δ*cpxR* strain, or Δ*cpxA*Δ*cpxR* strain (Fig. 2E). This verifies that each gene in the *escC–eseE* operon is under the positive regulation of CpxA.

### CpxA negatively regulates CpxR, and phosphorylated CpxR represses the transcription and expression of EseB

**Fig 3:**
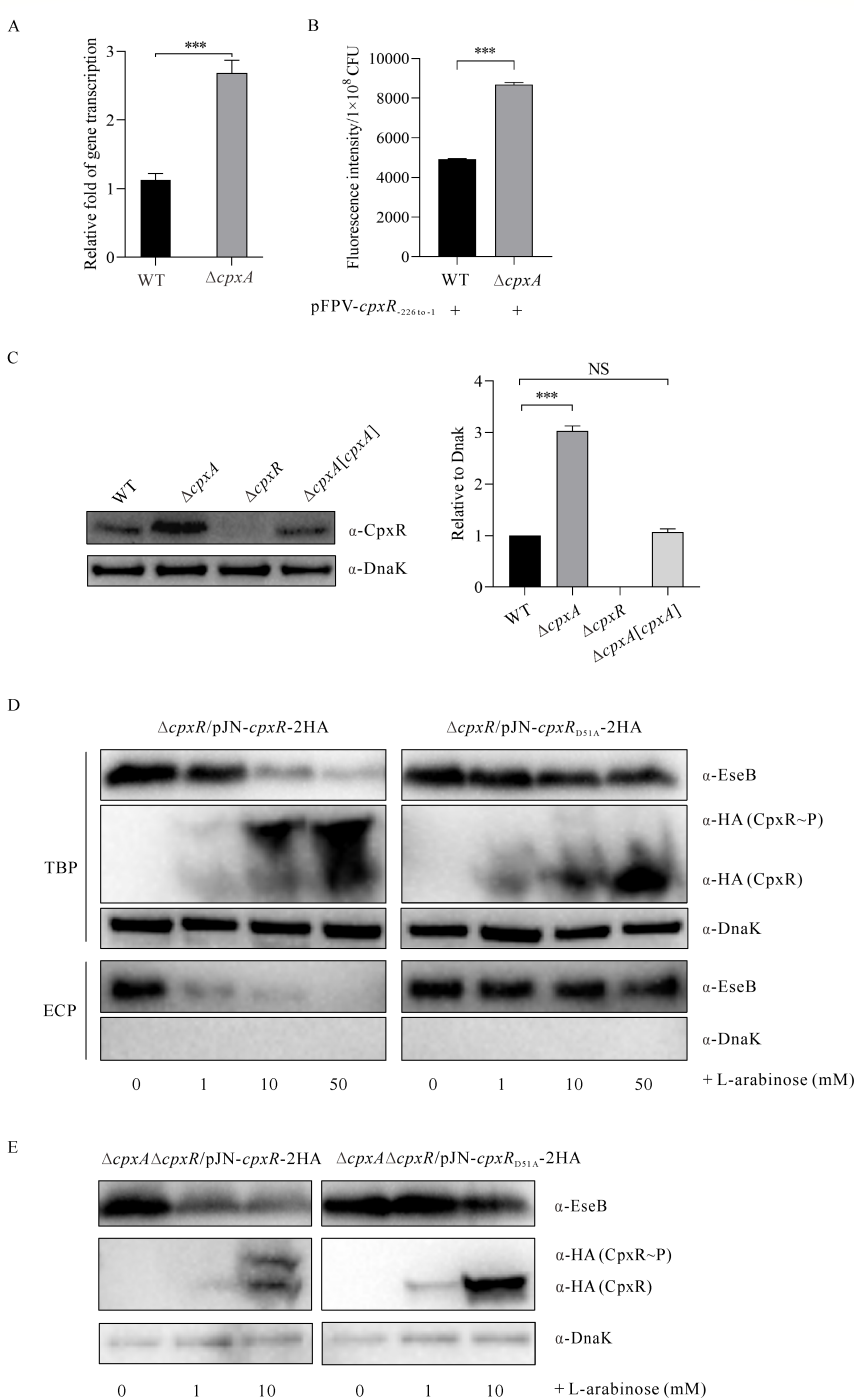
CpxA negatively regulates CpxR, and phosphorylated CpxR represses the transcription and expression of EseB. (**A**) The mRNA levels of *cpxR* in *E. piscicida* WT and ∆*cpxA* strains were examined by qRT-PCR. Multi-reference genes (*rpoB* and *gyrB*) were used to determine the relative transcript levels of *cpxR*. Data are expressed as mean ± SD. One-way ANOVA in SPSS was used to calculate the *P* values as compared to the WT strain. ***P < 0.001. (**B**) Fluorescence intensity of the GFP signal of the WT strain or of the ∆*cpxA* strain being introduced with pFPV25-*cpxR*_-226 to -1_-*gfp*. The GFP signal reflects the strength of the regulated *cpxR* promoter in different *E. piscicida* strains in DMEM at 24 hps. Data shown are representative of three independent experiments. ***P < 0.001. (**C**) Immunoblotting of the steady-state protein levels of CpxR in *E. piscicida* strains. Total bacterial proteins from similar amounts of *E. piscicida* strains were harvested at 24 hps in DMEM and probed with rabbit anti-CpxR and rabbit anti-DnaK antibodies (left panel). DnaK was used as a loading control. CpxR protein levels were quantified by densitometry and normalized to DnaK. The graphs show the relative ratios of intracellular CpxR, which are the averages of at least three independent experiments (right panel). ***P < 0.001; NS, not significant. (**D**) Immunoblotting for expression and secretion of EseB from *E. piscicida* strains ∆*cpxR/*pJN-*cpxR*-2HA and ∆*cpxR/*pJN-*cpxR*_D51A_-2HA. The TBPs and ECPs from equal amounts of *E. piscicida* strains were probed with rabbit anti-EseB, rabbit anti-HA (CpxR), and rabbit anti-DnaK antibodies. CpxR expression was induced by supplementation with L-arabinose at final concentrations of 1.0 mM, 10.0 mM, and 50.0 mM. To study the phosphorylated CpxR (HA), proteins were isolated by Mn^2+^ Phos-Tag SDS-PAGE gel electrophoresis before transfer to PVDF membrane. The images are representative of three independent experiments. (**E**) Immunoblotting of the steady-state protein levels of EseB, phosphorylated and non-phosphorylated CpxR-2HA in *E. piscicida* strains ∆*cpxA*∆*cpxR/*pJN-*cpxR*-2HA and ∆*cpxA*∆*cpxR/*pJN-*cpxR*_D51A_-2HA induced by L-arabinose supplementation at final concentrations of 1.0 mM and 10.0 mM. The TBPs from equal amounts of *E. piscicida* cultures were isolated by Mn^2+^ Phos-Tag SDS-PAGE gel electrophoresis to isolate the phosphorylated and non-phosphorylated CpxR-2HA (left panel) or CpxR_D51A_-2HA (right panel) before transfer to a PVDF membrane and probed with rabbit anti-EseB, rabbit anti-HA (CpxR), and rabbit anti-DnaK antibodies. The images are representative of three independent experiments.

How does CpxA regulate the *escC–eseE* operon, since CpxA is the sensor kinase of the TCS? To investigate this, the transcription of the response regulator CpxR was examined by qRT-PCR. Consistently, a threefold increase in CpxR transcription was detected in the Δ*cpxA* strain compared to the WT strain (Fig. 3A). To confirm this, pFPV-*cpxR*_-226 to -1_-*gfp* was introduced into WT and ∆*cpxA* strains to compare their GFP intensity. A significantly stronger GFP signal was detected in the ∆*cpxA* strain compared to the WT strain in DMEM at 24 hps (Fig. 3B). The GFP signal reflects the strength of the regulated *cpxR* promoter in different *E. piscicida* strains. This confirms that *cpxR* is negatively regulated by CpxA. Based on this evidence, the steady-state protein level of CpxR was examined. Consistently, a significantly increased steady-state protein level of CpxR was detected in the Δ*cpxA* strain compared to the WT strain or the Δ*cpxA*[*cpxA*] strain (Fig. 3C). These results demonstrate that CpxR is negatively regulated by CpxA and that CpxR accumulates in *E. piscicida* upon depletion of CpxA.

The 51st amino acid (aspartic acid, D) of CpxR was predicted as the phosphorylation site using Uniprot software (https://www.uniprot.org). For confirmation, pJN-*cpxR*_D51A_-2HA was constructed in which the 51st aspartic acid residue of CpxR was replaced by alanine (A). The pJN105 backbone is an arabinose-inducible expression vector (37). The Δ*cpxR* strain was then introduced with pJN-*cpxR*_D51A_-2HA or pJN-*cpxR*-2HA. It was observed that with increasing L-arabinose supplementation, the steady-state protein levels of phosphorylated CpxR (CpxR–*P*) and unphosphorylated CpxR increased. Meanwhile, the expression and secretion of EseB decreased sharply in the Δ*cpxR*/pJN-*cpxR*-2HA strain, but very slightly in the Δ*cpxR*/pJN-*cpxR*_D51A_-2HA strain (Fig. 3D). These results suggest that phosphorylated CpxR plays an important role in negatively regulating EseB transcription and expression.

What happens in the absence of CpxA, is CpxR still phosphorylated? To investigate this, pJN-*cpxR*-2HA or pJN-*cpxR*_D51A_-2HA was introduced into the Δ*cpxA*Δ*cpxR* strain. It was observed that with the increase in L-arabinose supplementation, both phosphorylated CpxR and non-phosphorylated CpxR increased, but the steady-state protein levels of EseB decreased sharply in the Δ*cpxA*Δ*cpxR*/pJN-*cpxR*-2HA strain (Fig. 3E, left panel). However, with the increase in L-arabinose supplementation, the steady-state protein levels of CpxR_D51A_ increase, whereas no phosphorylated CpxR_D51A_ was observed, and a slight decrease in EseB was detected in the Δ*cpxA*Δ*cpxR*/pJN-*cpxR*_D51A_-2HA strain (Fig. 3E, right panel). These data show that in the absence of CpxA, CpxR could still be phosphorylated by another kinase(s) via the 51st aspartic acid residue, resulting in reduced transcription and expression of EseB.

### The co-transcribed *cpxR* and *cpxA* are positively and directly regulated by phosphorylated CpxR, and to a lesser extent by unphosphorylated CpxR

**Fig 4:**
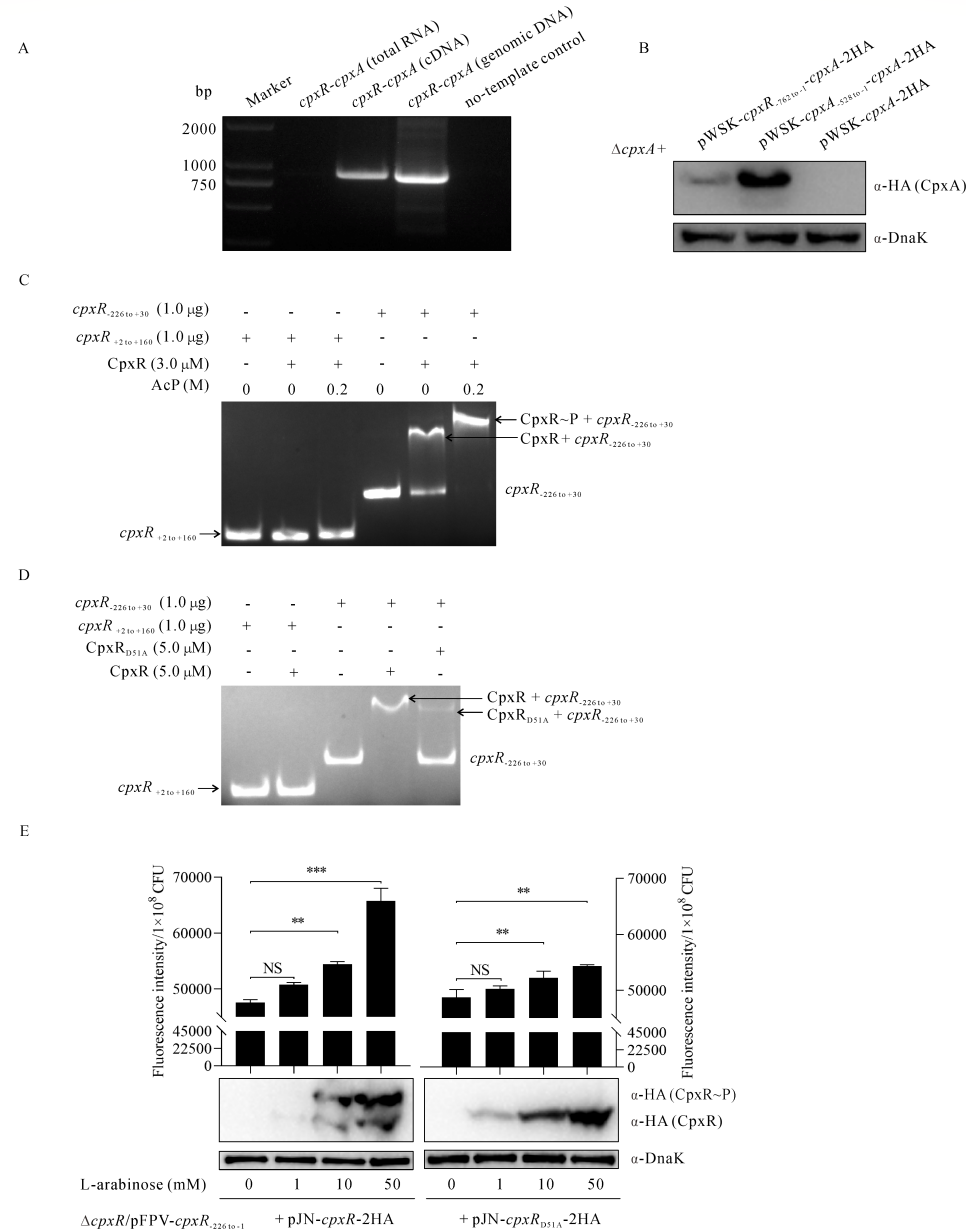
The co-transcribed *cpxR* and *cpxA* are positively and directly regulated by phosphorylated CpxR, and to a lesser extent by unphosphorylated CpxR. (**A**) The PCR products were electrophoresed and photographed. PCR was performed using the specific primer set covering *cpxR* and *cpxA*, with the total RNA, cDNA, or genomic DNA as the template. (**B**) Immunoblotting of the steady-state protein level of CpxA-2HA in *E. piscicida* strains. TBPs from equal amounts of WT/pWSK29-*cpxR*_-762 to -1_-*cpxA*-2HA strain (with the promoter upstream of *cpxR*), WT/pWSK29-*cpxA*_-528 to -1_-*cpxA*-2HA strain (with the promoter upstream of *cpxA*), and WT/pWSK29-*cpxA-*2HA strain (without the promoter) were probed with antibodies against HA (CpxA-2HA) and DnaK. The image shown is the representative of three independent experiments. (**C**) EMSA on phosphorylated CpxR and the DNA fragment nt −226 to +30 of *cpxR*. The Cy3-labeled DNA fragment (1.0 µg) was incubated with 3.0 µM CpxR, which was phosphorylated with 0.2 M lithium potassium acetyl phosphate (AcP) as a phosphate donor in the kinase buffer at 30.0°C for 1 h. The Cy3-labeled DNA fragment nt +2 to +160 of *cpxR* was used as a negative control probe. The protein-DNA complex was resolved on a 5% non-denaturing polyacrylamide gel. (**D**) EMSA on CpxR_D51A_ and the DNA fragment nt −226 to +30 upstream of *cpxR*. The Cy3-labeled DNA fragment (1.0 µg) was incubated with 5.0 µM CpxR_D51A_ or 5.0 µM CpxR (as the positive control). The Cy3-labeled DNA fragment nt +2 to +160 of *cpxR* was used as a negative control probe. The protein-DNA complex was resolved on a 5% non-denaturing polyacrylamide gel. (**E**) The fluorescence intensity of the GFP signal per 1 × 10^8^ CFU (colony-forming units) of the *E. piscicida* strain ∆*cpxR*/pFPV-*cpxR*_-226 to -1_-*gfp/*pJN-*cpxR*-2HA strain or ∆*cpxR*/pFPV-*cpxR*_-226 to -1_-*gfp*/pJN-*cpxR*_D51A_-2HA (top panel). Fluorescence intensity of GFP in *E. piscicida* was measured 24 h after subculture in DMEM supplemented with L-arabinose at a final concentration of 1.0 mM, 10.0 mM, or 50.0 mM. Mn^2+^ Phos-Tag SDS-PAGE gel electrophoresis was used to isolate the phosphorylated and non-phosphorylated CpxR-2HA (left panel) or CpxR_D51A_-2HA (right panel) before transfer to a PVDF membrane and probed with anti-HA and DnaK antibodies. DnaK was used to indicate similar amounts of protein loading per lane. Images shown are representative of three independent experiments.

Is *cpxA* transcribed independently or co-transcribed with *cpxR*? To investigate whether *cpxR* and *cpxA* are on the same transcription chain, the primer pair *cpxR-cpxA*-for (located in *cpxR*) and *cpxR-cpxA-*rev (located in *cpxA*) was used to amplify the degenomicized total cDNA of *E. piscicida* PPD130/91. No bands were detected when total RNA or distilled water was used as the template, whereas bands of similar size were obtained when cDNA or genomic DNA was used as the template (Fig. 4A). This indicates that *cpxR* and *cpxA* are co-transcribed as a single operon. The *cpxA* gene is located downstream of *cpxR*. Does *cpxA* have its own promoter? To investigate this, the promoterless low-copy expression plasmid pWSK29 was inserted with *cpxA* together with the promoter upstream of *cpxR* (pWSK-*cpxR*_-762 to -1_-*cpxA*-2HA), with *cpxA* with the promoter upstream of *cpxA* (pWSK-*cpxA*_-528 to -1_-*cpxA*-2HA) or with *cpxA* without the promoter (pWSK-*cpxA*-2HA) before being introduced into the Δ*cpxA* strain to examine the steady-state protein levels of CpxA-2HA by immunoblotting. The highest protein level of CpxA-2HA was detected in the Δ*cpxA*/pWSK-*cpxA*_-528 to -1_-*cpxA*-2HA strain, the intermediate level from the Δ*cpxA*/pWSK-*cpxR*_-762 to -1_-*cpxA*-2HA strain, and none from the Δ*cpxA*/pWSK-*cpxA*-2HA strain (Fig. 4B). This indicates that *cpxA* is mainly transcribed under its own promoter and, to a lesser extent, co-transcribed with *cpxR* under the *cpxR* promoter.

Which regulator(s) control(s) the *cpxR–cpxA* operon? Based on previous reports in *Escherichia coli* and *Y. pseudotuberculosis* (38, 39), a conserved binding motif (5′-GTAACttcagGTAAT-3′) of CpxR was found upstream of the *cpxR–cpxA* operon. Lithium potassium acetyl phosphate (AcP) was used as a donor to phosphorylate CpxR-His_6_ *in vitro* as described by León-Montes et al. (27). The full-length CpxR-His_6_, AcP, and a Cy3-labeled DNA fragment spanning nt −226 to +30 of *cpxR* were used for electrophoretic mobility shift assay (EMSA). The mobility of 1.0 µg DNA fragment nt −226 to +30 of *cpxR* was partially shifted in the presence of 3.0 µM CpxR-His_6_, whereas a complete shift occurred when CpxR-His_6_ was phosphorylated by AcP (CpxR–*P*). As a negative control, 1.0 µg Cy3-labeled DNA fragment nt +2 to +160 of *cpxR* did not shift with either form of CpxR-His_6_ (Fig. 4C). These results indicate that phosphorylated CpxR-His_6_ (CpxR–*P*) can bind to the *cpxR* promoter more efficiently than the CpxR-His_6_ that was not phosphorylated by AcP. Does CpxR_D51A_ still bind to the promoter of *cpxR*? To test this, CpxR_D51A_-His_6_ was expressed and purified. The 5.0 µM CpxR_D51A_-His_6_ or CpxR-His_6_ protein (as the positive control) was subjected to EMSA assays with 1.0 µg Cy3-labeled DNA fragment nt −226 to +30 of *cpxR*. Partial binding between CpxR_D51A_-His_6_ and the *cpxR* promoter was detected when complete binding between CpxR-His_6_ and the *cpxR* promoter occurred (Fig. 4D). These results confirm that the site-mutated version of CpxR (CpxR_D51A_) is still able to bind to the *cpxR* promoter, although much less efficiently than wild-type CpxR.

How does CpxR regulate the *cpxR–cpxA* operon, positively or negatively? To investigate this, pFPV-*cpxR*_-226 to -1_-*gfp* was constructed and introduced into the Δ*cpxR*/pJN-*cpxR*-2HA and Δ*cpxR*/pJN-*cpxR*_D51A_-2HA strains. With increasing L-arabinose supplementation, the steady-state protein levels of either unphosphorylated CpxR or phosphorylated CpxR (CpxR–*P*) increase, and the intensity of the GFP signal increases strongly. However, the intensity of the GFP signal increased slightly when the expression of CpxR_D51A_ was induced, in which case phosphorylation of CpxR_D51A_ was not detectable (Fig. 4E). This shows that the phosphorylated CpxR binds directly and efficiently to the *cpxR* promoter and positively regulates the *cpxR–cpxA* operon, while CpxR that has not been subjected to phosphorylation by AcP or the site-mutated version of CpxR (CpxR_D51A_) is still able to bind to the *cpxR* promoter and regulate the *cpxR–cpxA* operon, albeit to a lesser extent.

Together, the *cpxR* and *cpxA* genes are independently transcribed, in addition to being co-transcribed, and the *cpxR–cpxA* operon is directly and positively regulated by CpxR.

### CpxAR is directly regulated by another TCS EsrAB

**Fig 5:**
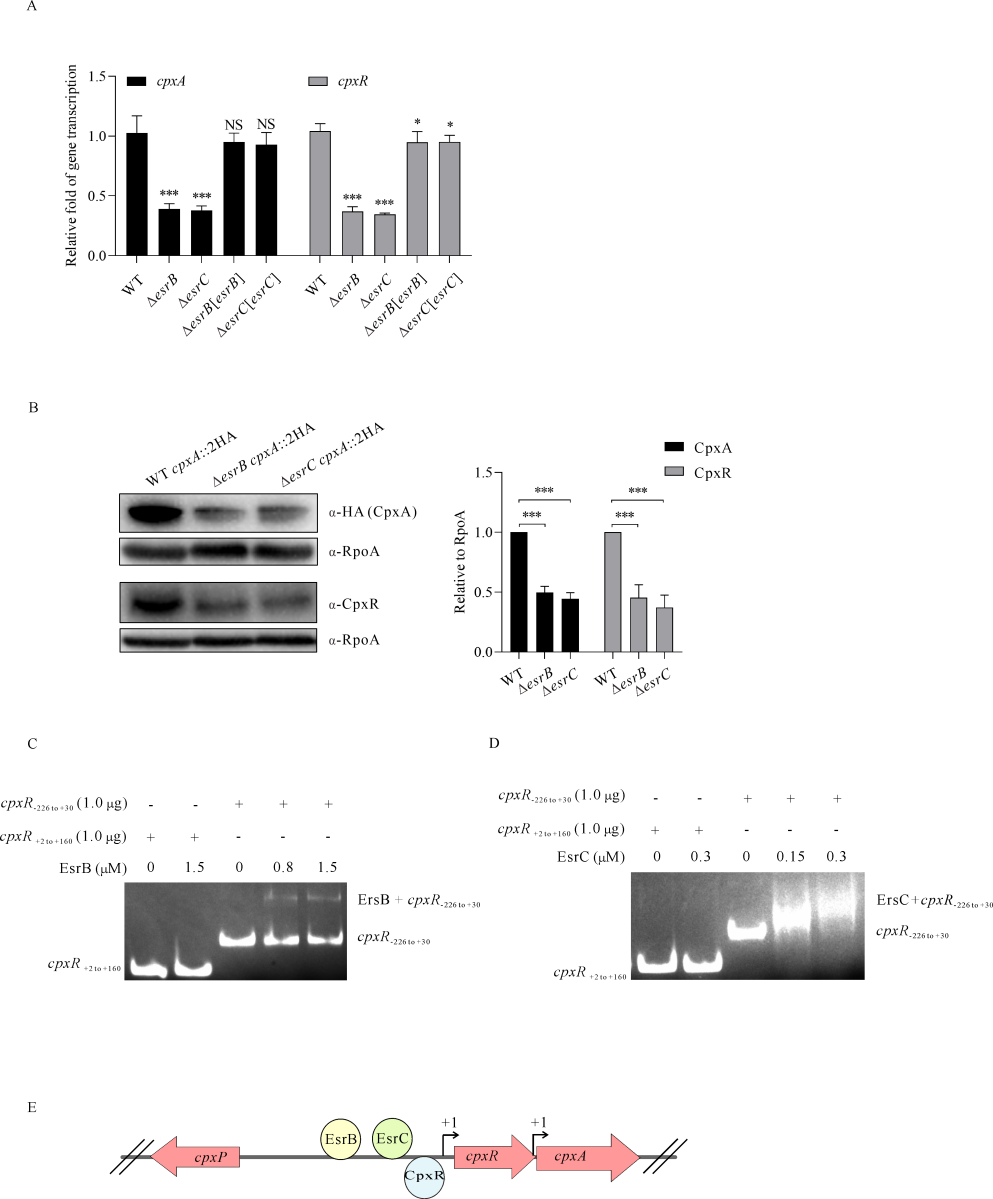
EsrB and EsrC bind to upstream of *cpxR* and positively regulate the *cpxR–cpxA* operon. (**A**) The transcript levels of *cpxR* and *cpxA* in *E. piscicida* strains were analyzed by qRT-PCR. Transcription of *cpxR* or *cpxA* was normalized against the multi-reference genes *rpoB* and *gyrB.* Means ± SD of one representative experiment are shown. One-way ANOVA in SPSS was used to calculate the *P* values as compared to the WT strain. ***P < 0.001; NS, not significant. (**B**) Immunoblotting of the steady-state protein levels of CpxR or CpxA-2HA in *E. piscicida* strains. TBPs of WT, ∆*esrB,* and ∆*esrC* strains were resolved and probed with rabbit anti-CpxR and rabbit anti-HA (CpxA-2HA), respectively. RpoA was included to show the similar amount of protein loading per lane (left panel). Protein levels of CpxA-2HA and CpxR were quantified by densitometry and normalized to RpoA. The graphs show the relative ratios of intracellular CpxR or CpxA-2HA, which are the average of at least three independent experiments (right panel); ***P < 0.001. (**C**) EMSA on EsrB or EsrC and the DNA fragment nt −226 to +30 of *cpxR*. The Cy3-labeled DNA fragment −226 to +30 upstream of *cpxR* (1.0 µg) or +2 to +160 of *cpxR* (negative control) was incubated with the indicated concentrations of EsrB or EsrC protein for EMSA before the protein-DNA complex was resolved on a 5.0% non-denaturing polyacrylamide gel. (**D**) Genetic organization of the *cpxR–cpxA* operon in *E. piscicida.* The *cpxR* and *cpxA* share the same promoter (−226 to −1 upstream of *cpxR*), but *cpxA* could also be transcribed independently. (E) The *cpxP* gene is located in a separate operon next to the *cpxA–cpxR* operon, which is directly and positively regulated by CpxR, EsrB, and EsrC.

TCSs may be interconnected to coordinately regulate virulence and biofilm formation in bacterial pathogens. T3SS in *E. piscicida* is tightly and positively regulated by the TCS EsrAB (28). Since CpxAR regulates the *escC–eseE* operon of the T3SS gene cluster, is the transcription of the *cpxR–cpxA* operon under the control of EsrAB? To investigate this, the low-copy plasmid pWSK29 was used to express EsrB or EsrC *in trans* to complement the ∆*esrB* strain or ∆*esrC* strain, respectively. And the transcript levels of *cpxA* and *cpxR* in WT, ∆*esrB*, ∆*esrC*, Δ*esrB*[*esrB*], and Δ*esrC*[*esrC*] strains were determined by qRT-PCR assay. It was found that the transcript of either *cpxA* or *cpxR* dramatically decreased in the absence of EsrB or EsrC compared to the WT strain and was restored in the complementary strains (Fig. 5A). Consistently, the steady-state protein levels of CpxA-2HA or CpxR in the Δ*esrB* strain or Δ*esrC* strain were also much lower than in the WT strain when similar amounts of proteins were loaded, as indicated by RpoA (Fig. 5B). Furthermore, either EsrB-His_6_ protein (full length) or EsrC-His_6_ protein (full length) was able to bind to nt −226 to +30 of *cpxR* as shown by EMSA (Fig. 5C). These results demonstrate that the *cpxR–cpxA* operon is under direct and positive regulation by EsrB and EsrC (Fig. 5D).

### CpxAR and EsrAB, together with EseE, coordinately regulate biofilm formation in *E. piscicida*

**Fig 6:**
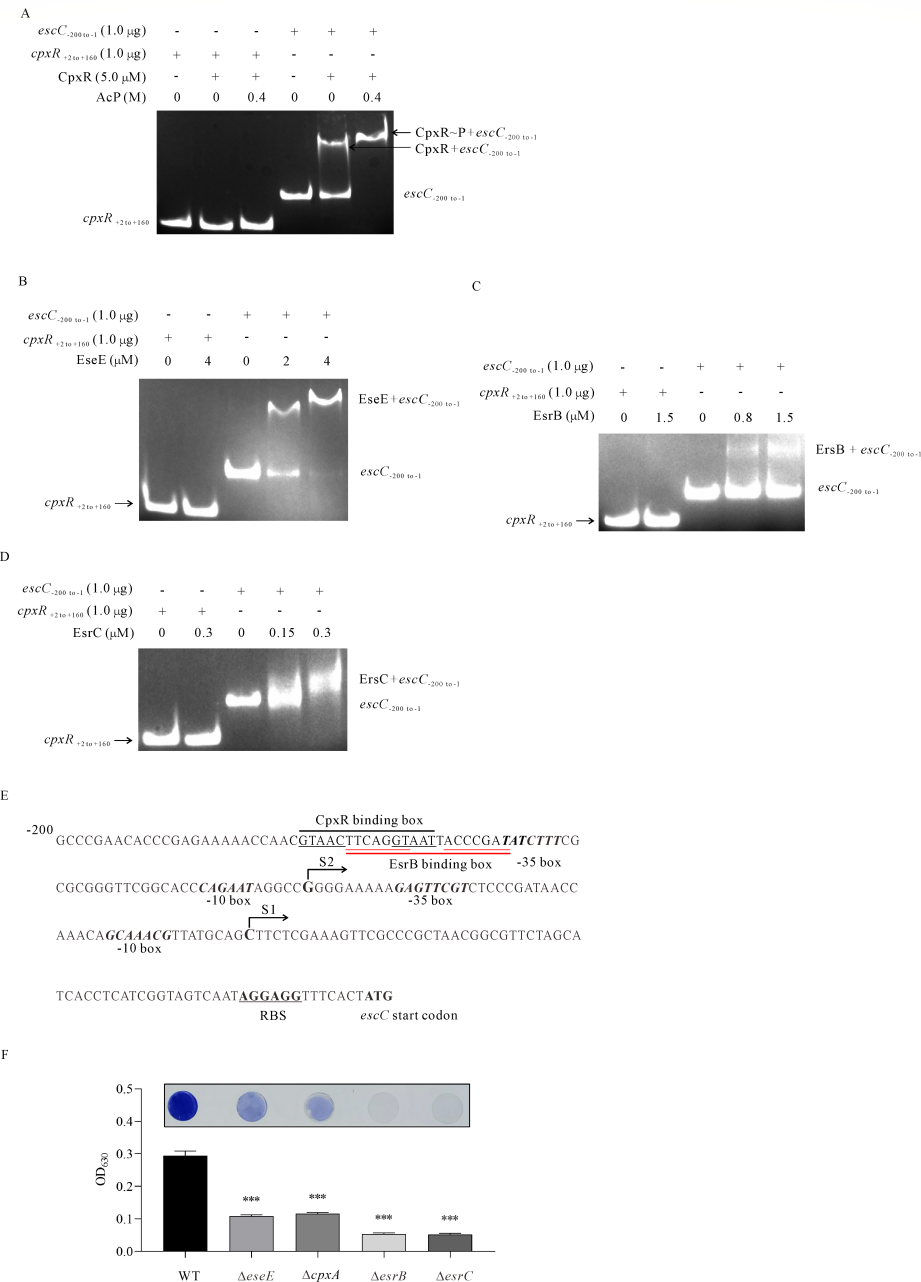
CpxR, EsrB, EsrC, and EseE coordinately regulate biofilm formation by directly regulating the *escC–eseE* operon, in which EseB is encoded. (**A**) EMSA on phosphorylated and non-phosphorylated CpxR and the Cy3-labeled DNA fragment nt −200 to −1 upstream of *escC*. The CpxR protein (5.0 µM) was incubated with or without 0.4 M lithium potassium acetyl phosphate (AcP) as the phosphate donor in the kinase buffer at 30.0°C for 1 h before the mixture was added to the Cy3-labeled DNA fragment from −200 to −1 upstream of *escC* (1.0 µg) or +2 to +160 of *cpxR* (negative control) for EMSA. Protein-DNA complexes were resolved on a 5% non-denaturing polyacrylamide gel. (**B**) EMSA between the indicated concentrations of EseE and the Cy3-labeled DNA fragment from −200 to −1 upstream of *escC* (1.0 µg) or +2 to +160 of *cpxR* (negative control) before resolving the protein-DNA complex on a 5.0% non-denaturing polyacrylamide gel. (**C**) EMSA between the indicated concentrations of EsrB and the Cy3-labeled DNA fragment from −200 to −1 of *escC* (1.0 µg) or +2 to +160 of *cpxR* (negative control) before resolving the protein-DNA complex on a 5.0% non-denaturing polyacrylamide gel. (**D**) EMSA between the indicated concentrations of EsrC and the Cy3-labeled DNA fragment of −200 to −1 of *escC* (1.0 µg) or +2 to +160 of *cpxR* (negative control) before resolving the protein-DNA complex on a 5.0% non-denaturing polyacrylamide gel. (**E**) The promoter region of *escC*. The CpxR box and the EsrB box, where CpxR or EsrB binds to the *escC* promoter, are underlined. S1 and S2 are the predicted transcription start sites. The bold italic nucleotide motifs indicate the −35 box and the −10 box of the two promoters; the predicted RBS and the *escC* start codon are also labeled. (**F**) Biofilm formation in *E. piscicida* WT, ∆*eseE*, ∆*cpxA*, ∆*esrB,* and ∆*esrC* strains. *E. piscicida* strains were subcultured for 24 h in DMEM in a 24-well plate horizontally embedded with coverslips, and the biofilm developed on the coverslips was fixed and stained with 0.2% crystal violet. The images shown are representative of three independent experiments (top panel), and biofilm formation was assessed by examining the OD_630_ of the dissolved crystal violet (bottom panel). ***P < 0.001.

Our previous research showed that EsrB, EsrC, and free EseE promote biofilm formation by positively regulating *eseB* in *E. piscicida* (4). It remains unclear whether the *escC–eseE* operon is under the direct regulation of these regulators or not. To investigate this, the binding between the Cy3-labeled DNA fragment nt −200 to −1 upstream of *escC* (*escC*_-200 to -1_) and EseE-His_6_, EsrB-His_6_, EsrC-His_6_, and CpxR-His_6_ was examined by EMSA. It was found that CpxR-His_6_ phosphorylated by AcP binds to the promoter of *escC* and causes a complete DNA shift, whereas CpxR-His_6_ not treated with AcP can still bind, but causes a partial DNA shift (Fig. 6A). Each of EseE, EsrB, and EsrC could directly regulate the *escC–eseE* operon. Specifically, 4.0 µM EseE, 1.5 µM EsrB, or 0.3 µM EsrC caused a partial DNA shift (Fig. 6B through D). As a negative control, 1.0 µg Cy3-labeled DNA fragment nt +2 to +160 of *cpxR* did not shift with any of the proteins examined. Together, CpxR, EseE, EsrB, and EsrC directly regulate the *escC–eseE* operon to control the transcription and expression of EseB in *E. piscicida*.

Upstream of *escC*, two sets of promoter regions of −35 box and −10 box were predicted at https://molbiol-tools.ca/Promoters.htm#opennewwindow. By 5′-RACE assay, two transcription start sites (TSS) were identified upstream of *escC*, one being the S1 site at −69 bp (C) and the other being the S2 site at −118 bp (G), when taking the *escC* translation start site as +1. Based on previous reports of the conserved binding motif of CpxR in *E. coli* and in *Y. pseudotuberculosis* (38, 39) and on the conserved binding motif of EsrB in *E. piscicida* (40), the conserved binding motif of CpxR 5′-GTAACttcagGTAAT-3′ and the conserved binding motif of EsrB 5′-TTCAGGTaattACCCGAT-3′ were found and labeled in the promoter region of the *escC−eseE* operon (Fig. 6E).

Biofilm formation in Δ*eseE*, Δ*cpxA*, Δ*esrB,* and Δ*esrC* strains was then examined by crystal violet staining at 24 hps in DMEM. It was observed that deletion of *esrB* or *esrC* almost abolished biofilm formation, whereas depletion of EseE or CpxA moderately but significantly attenuated biofilm formation (Fig. 6F). These results indicate that CpxR, EseE, EsrB, and EsrC directly regulate the transcription of the *escC*−*eseE* operon, thereby tightly controlling biofilm formation in *E. piscicida*.

### Indole reduces biofilm formation in *E. piscicida*

**Fig 7:**
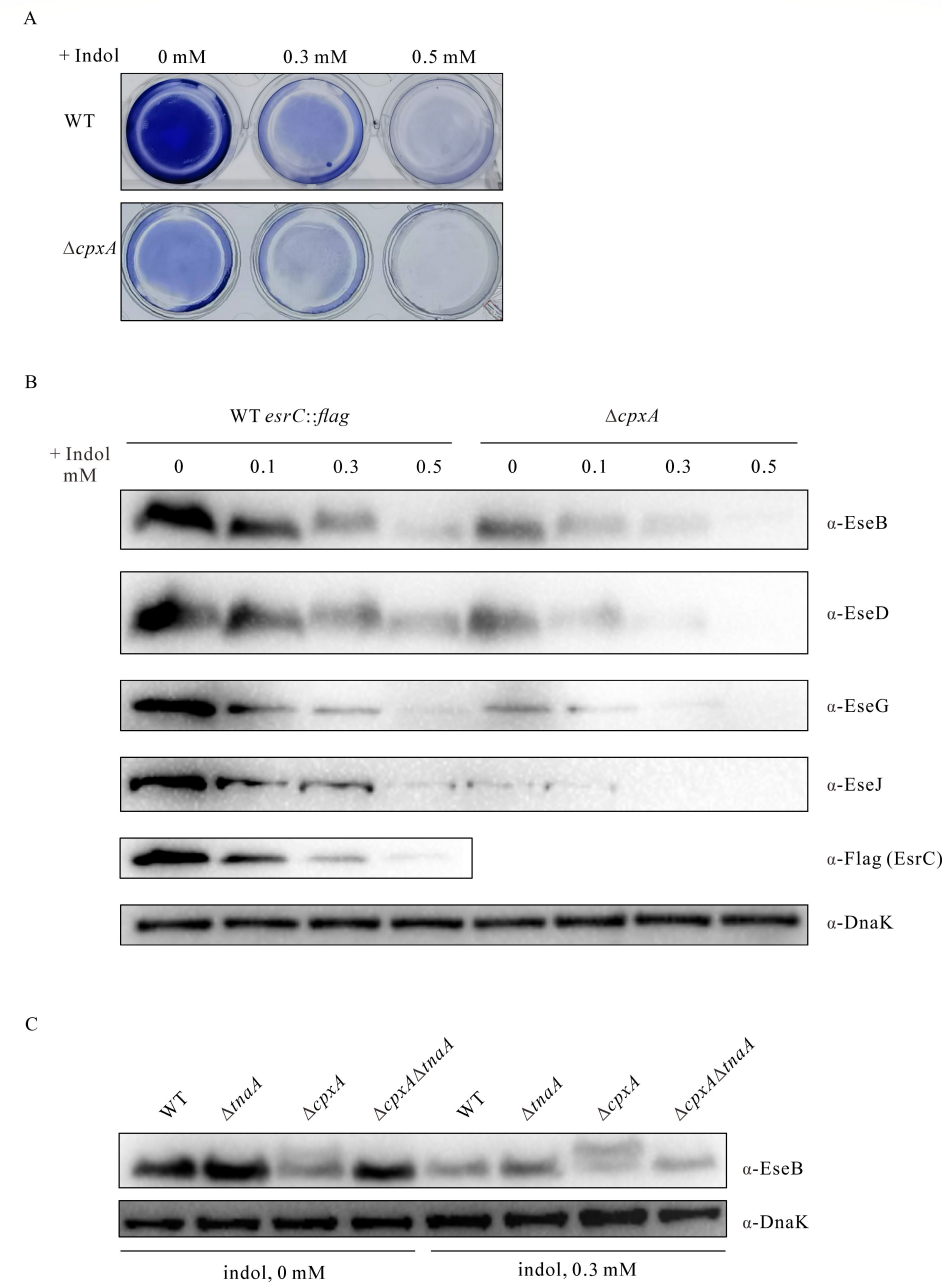
Both the exogenous and endogenous indole suppressed biofilm formation in *E. piscicida*. (**A**) Biofilm formed by *E. piscicida* in the presence of exogenous indole. WT and ∆*cpxA* strains subcultured in DMEM in a 24-well plate were supplemented with indole at final concentrations of 0 mM, 0.3 mM, and 0.5 mM. After 24 hours, the biofilm formed on the bottom of the plate was fixed and stained with 0.2% crystal violet. Images are representative of at least three independent experiments. (**B**) Immunoblotting of the steady-state protein levels of EseB, EseD, EseG, EseJ, and EsrC-FLAG in *E. piscicida* strains subcultured for 24 h in DMEM in the presence of 0.1 mM, 0.3 mM, and 0.5 mM indole. TBPs from similar amounts of *E. piscicida* strains were probed with rabbit anti-EseB, rabbit anti-EseD, rabbit anti-EseG, rabbit anti-EseJ, rabbit anti-DnaK, and mouse anti-FLAG (EsrC-FLAG) antibodies. Images shown are representative of at least three independent experiments. (**C**) Immunoblotting of the steady-state protein levels of EseB in *E. piscicida* WT, ∆*tnaA*, ∆*cpxA*, and ∆*tnaA*∆*cpxA* strains subcultured for 24 h in DMEM in the presence of 0.3 mM indole. Similar amounts of bacterial lysates from each strain were probed with rabbit anti-EseB and rabbit anti-DnaK antibodies. Images shown are representative of at least three independent experiments.

Indole is one of the microbiota-derived chemical signals present in the gut lumen and is also produced by *E. piscicida* itself (31, 34). To determine the contribution of indole to biofilm formation, *E. piscicida* WT and ∆*cpxA* strains were cultured in the presence of 0.3 mM and 0.5 mM indole. Biofilm formation was found to be inversely proportional to indole supplementation as shown by crystal violet staining of the biofilm (Fig. 7A). The steady-state protein levels of EseB, EseD, EseG, and EseJ decreased with the increasing indole supplementation in either the WT *esrC::flag* strain or the Δ*cpxA* strain when similar amounts of proteins were loaded (Fig. 7B). This indicates that the expression of T3SS proteins in *E. piscicida* is inversely proportional to exogenous indole supplementation.

*E. piscicida* produces indole by metabolizing tryptophan via the tryptophanase TnaA (31). Deletion of *tnaA* slightly increased the steady-state protein level of EseB, and further deletion of *cpxA* reduced EseB to levels similar to the WT strain (Fig. 7C). This suggests that endogenous indole also inhibits EseB expression. Supplementation with 0.3 mM indole reduced EseB expression in every strain examined (Fig. 7C). These results suggest that indole, either produced by *E. piscicida* itself or released by the microbiota, suppresses biofilm formation by downregulating the expression of the T3SS needle tip protein EseB.

## DISCUSSION

The community living within the biofilm significantly improves bacterial tolerance to stresses such as host-derived killing, antibiotics, etc. (10, 41). Two-component systems play an important role in the regulation of biofilm formation. Here we have shown that the histidine kinase CpxA regulates biofilm formation in *E. piscicida* by negatively regulating its response regulator CpxR. CpxR binds directly to the promoter region of the *escC−eseE* operon to repress, whereas EsrB, EsrC, and EseE bind to the same promoter region to promote transcription and expression of EseB. In addition, either endogenous or exogenous indole inhibits EseB filament-mediated biofilm formation in *E. piscicida*.

The histidine kinase CpxA plays a dual role, acting either as a kinase transferring phosphate to CpxR or as a phosphatase removing phosphate from CpxR−*P* (17). When not stimulated to autophosphorylate, CpxA acts as a phosphatase of its cognate response regulator CpxR−*P*, and the balance between kinase and phosphatase activities modulates the output response (17). In addition to CpxA, CpxR can also be phosphorylated by the AckA-Pta pathway, which generates acetyl phosphate from acetyl coenzyme A (acetyl-CoA) using the enzymes phosphotransacetylase (Pta) and acetate kinase (AckA), and the phosphoryl group from the acetyl phosphate obtained is then transferred to CpxR (21, 23). In *Y. pseudotuberculosis*, CpxR is phosphorylated mainly through the AckA-Pta pathway, and increased CpxR−*P* represses the Ysc-Yop T3SS (25). Avian pathogenic *Escherichia coli* also produces CpxR−*P* through the AckA-Pta pathway in the ∆*cpxA* strain, and increased CpxR−*P* suppresses its biofilm formation (17). Consistently, CpxR expressed by pJN-*cpxR*-2HA in the ∆*cpxA*∆*cpxR* strain could also be phosphorylated and inhibit the expression of the T3SS protein EseB. The CpxR was speculated to be phosphorylated via the AckA-Pta pathway, since AckA protein (NCBI reference sequence: WP_012849226.1) and Pta protein (NCBI reference sequence: WP_034167896.1) are present in *E. piscicida*. By contrast, in enterohemorrhagic *Escherichia coli* (EHEC), upon activation by the adhesion signal, CpxA phosphorylates CpxR, and subsequently CpxR−*P* binds to the *lrhA* promoter to upregulate LrhA, which, in turn, upregulates the T3SS needle tip protein EspA (42). EspA is the component of a filamentous surface organelle, the “EspA filament,” which mediates biofilm formation in EHEC (43). Since the assembly of EseB filaments on the surface of *E. piscicida* alters envelope integrity, CpxR may have played a key role in envelope integrity by negatively regulating the *escC−eseE* operon. It is speculated that by repressing EseB expression and EseB filament assembly, CpxAR maintains the stability and integrity of the *E. piscicida* envelope.

TCSs play a key role in sensing environmental signals or stimuli and regulating their downstream genes to adapt to changes (44). *E. piscicida* EsrAB shares homology with *Salmonella* SsrAB and plays an important role not only in the regulation of T3SS but also in cell signal transduction (19, 28). CpxAR represses *Salmonella* Pathogenicity Island 2 (SPI-2) by acting directly on the *ssrA−ssrB* operon (27). In *E. piscicida*, EsrB or EsrC binds to the promoter of the *cpxR−cpxA* operon and positively regulates CpxAR. It is proposed that EsrAB and CpxAR coordinate in the regulation of biofilm formation in *E. piscicida* by sensing and responding to environmental cues.

Indole, a microbial metabolite of tryptophan, suppresses EHEC adhesion to intestinal epithelial cells, thereby inhibiting biofilm formation (45). Indole attenuates *Salmonella* invasion by inhibiting *Salmonella* Pathogenicity Island 1 (SPI-1), and its roles in the gastrointestinal tract are partially exerted in a PhoQP-dependent manner (46). CpxA has been identified as an indole sensor in EHEC that downregulates the expression of LEE (locus of enterocyte effacement) in the intestinal compartment (31). However, CpxA does not appear to act as an indole sensor in *E. piscicida*, as indole supplementation strongly reduces the steady-state protein level of EseB in the ∆*cpxA*∆*tnaA* strain. This is in stark contrast to the observation in EHEC where the ∆*cpxA*∆*tnaA* strain does not respond to indole supplementation (31). Similar to EHEC, both endogenous and exogenous indole suppress T3SS in *E. piscicida*. The TCS PhoQP in *E. piscicida* senses changes in environmental temperature and Mg^2+^ concentration and regulates T3SS through direct activation of EsrB (47). It is speculated that PhoQP or EsrAB may be involved in indole sensing in *E. piscicida*, but this requires further study.

**Fig 8:**
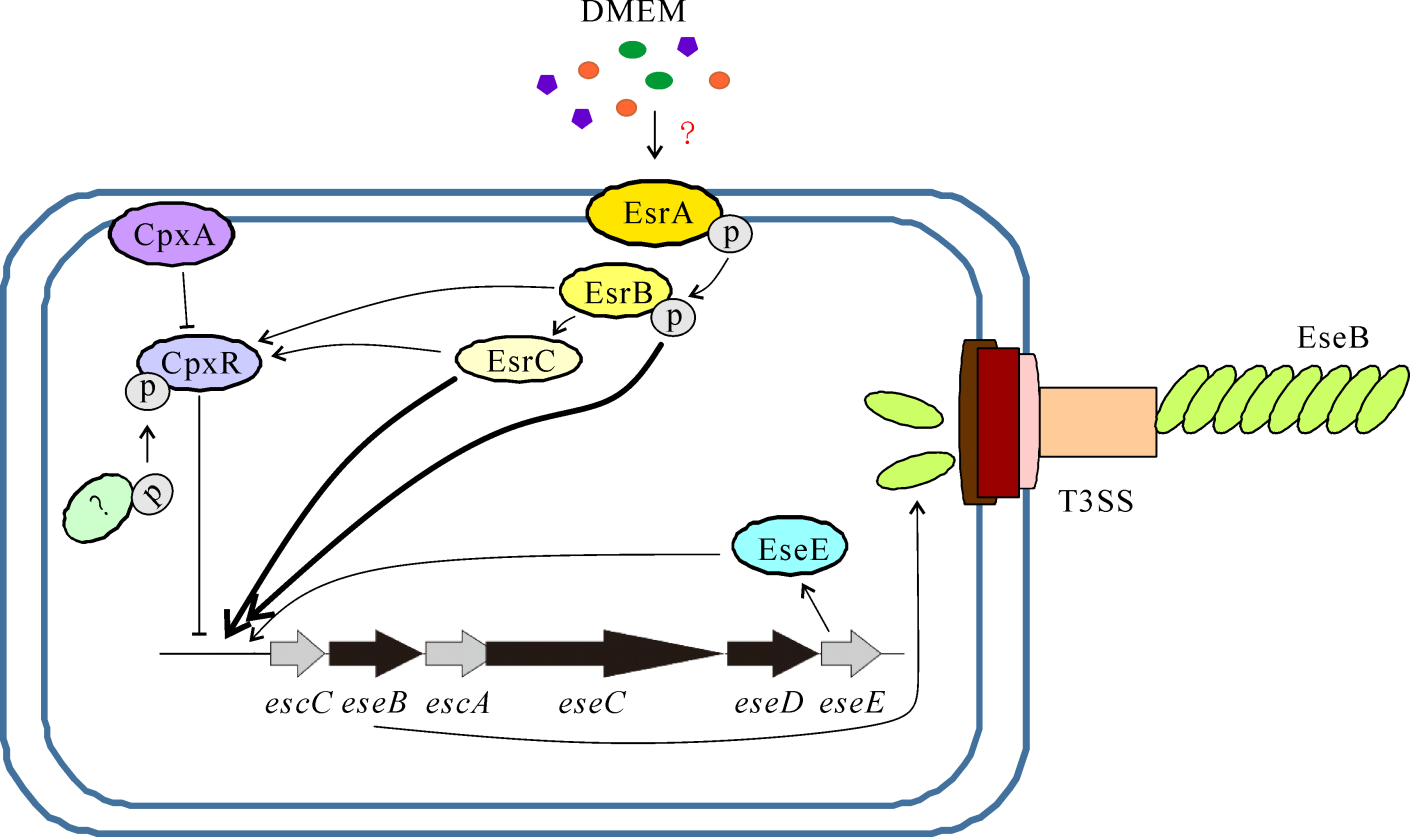
Schematic representation of the regulation of biofilm formation in *E. piscicida*. Culturing *E. piscicida* in DMEM simulates *in vivo* nutrient limitation conditions that activate T3SS. Increased expression of the T3SS needle tip protein EseB promotes EseB-filament-mediated bacterial-bacterial interaction and biofilm formation. CpxA negatively regulates CpxR, whereas EsrB, EsrC, and CpxR directly and positively regulate CpxR. Phosphorylated CpxR binds to the promoter of the *escC−eseE* operon to negatively regulate *eseB*. EseE, EsrB, or EsrC binds to the promoter of the *escC−eseE* operon to positively regulate *eseB*. EsrB and EsrC, which play a major role in this regulation, are indicated by the thick arrow. Microbiota-derived indole, sensed and transduced by an unknown signaling pathway, downregulates EseB filament-mediated biofilm formation.

In conclusion, the histidine kinase CpxA controls biofilm formation in *E. piscicida* through negative regulation of its response regulator CpxR. Phosphorylated CpxR (CpxR−*P*) binds directly and efficiently to the promoter region of the *escC−eseE* operon and negatively regulates it, whereas EsrB, EsrC, and EseE each bind directly to the promoter region of the *escC−eseE* operon, where EseB is encoded, but positively regulate it. By regulating T3SS, *E. piscicida* could control biofilm formation in response to environmental cues such as indole or DMEM components (Fig. 8). It is suggested that some components in DMEM, metabolites of *E. piscicida*, or indole are sensed by PhoQ or EsrA, which upregulate T3SS through the response regulator EsrB. The response regulator PhoP partly regulates T3SS through EsrB (19). In addition, the response regulator EsrB or the regulator EsrC directly and positively regulates CpxR. It is speculated that the change in the bacterial envelope caused by the assembly of the EseB filament is sensed by CpxA, which, in turn, downregulates the *escC−eseE* operon through its response regulator CpxR to maintain the integrity of the bacterial envelope. Understanding the regulatory pathways and environmental factors involved in biofilm formation is essential for developing effective strategies to prevent edwardsiellosis through biofilm control.

## MATERIALS AND METHODS

### Bacterial strains and culture conditions

The bacterial strains and plasmids used in this study are described in Table 1. *Edwardsiella piscicida* PPD130/91 (48) and its derivative strains were grown statically in tryptic soy broth (TSB; BD Biosciences) at 28°C. To activate T3SS, *E. piscicida* strains were grown in Dulbecco’s modified Eagle’s medium (DMEM; Invitrogen) at 25°C under 5.0% (vol/vol) CO_2_. The medium was supplemented as required with the appropriate antibiotics at the following concentrations: 12.5 µg/mL colistin (Col; Sigma), 34.0 µg/mL chloramphenicol (Cm; Amresco), 50.0 µg/mL gentamicin (Gm; Amresco), 50.0 µg/mL kanamycin (Km; Solarbio), and 100.0 µg/mL ampicillin (Amp; Sigma).

**TABLE 1:** Strains and plasmids used in this study^*a*^

Strain or plasmid	Description	Reference or source
*E. pisccida* strains		
PPD130/91	Wild type; Col^r^	(48)
∆*cpxA*	PPD130/91, *cpxA* in-frame deletion of aa 76 to 1,080	This study
∆*cpxR*	PPD130/91, *cpxR* in-frame deletion of aa 76 to 492	This study
∆*cpxA*∆*cpxR*	PPD130/91, in-frame deletion of *cpxA* and *cpxR*	This study
Δ*tnaA*	PPD130/91, *tnaA* in-frame deletion of aa 1 to 1,416	This study
Δ*cpxA*Δ*tnaA*	PPD130/91, in-frame deletion of *cpxA* and *tnaA*	This study
Δ*cpxA*[*cpxA*]	Δ*cpxA* with pWSK-*cpxR*_−762 to −1_-*cpxA*-2HA; Col^r^, Amp^r^	This study
Δ*cpxR*[*cpxR*]	Δ*cpxR* with pWSK-*cpxR*_−365 to −1_-*cpxR*-2HA; Col^r^, Amp^r^	This study
∆*cpxA*∆*cpxR*[*cpxAcpxR*]	∆*cpxA*∆*cpxR* with pWSK-*cpxR*_−365 to −1_-*cpxR-cpxA*-2HA; Col^r^, Amp^r^	This study
∆*eseE*	PPD130/91, *eseE* in-frame deletion of aa 4 to 119	(6)
∆*esrB*	PPD130/91, *esrB* in-frame deletion of aa 82 to 597	(5)
∆*esrC*	PPD130/91, *esrC* in-frame deletion of aa 12 to 220	(28)
Δ*esrB*[*esrB*]	Δ*cpxA* with pWSK-*esrB*_-610 to -1_-*esrB*-FLAG; Col^r^, Amp^r^	This study
Δ*esrC*[*esrC*]	Δ*cpxA* with pWSK-*esrC*_-420 to -1_-*esrC*-FLAG; Col^r^, Amp^r^	(49)
WT *cpxA*::2 HA	PPD130/91 with chromosomal expression of CpxA-2HA; Col^r^, Km^r^	This study
∆*esrB cpxA*::2 HA	∆*esrB* with chromosomal expression of CpxA-2HA; Col^r^, Km^r^	This study
∆*esrC cpxA*::2 HA	∆*esrC* with chromosomal expression of CpxA-2HA; Col^r^, Km^r^	This study
WT *esrC*::*flag*	PPD130/91 with chromosomal expression of EsrC-FLAG; Col^r^	This study
*E. coli* strains		
DH_5α_	α complementation	Stratagene
MC1061(*λpir*)	*thi thr-1 leu6 proA2 his-4 argE2 lacY1 galK2 ara14 xyl5 supE44*, λ *pir*	Stratagene
S17-1(*λpir*)	RK2 *tra* regulon, *pir*, host for *pir*-dependent plasmid	Stratagene
BL21(DE3)/pLysS	F^－^ *ompT hsdS* (r_B_^－^m_B_^－^) *gal dcm* (DE3) *tonA* pLysS (Cm^r^)	Invitrogen
DE3/pET-*cpxR*	BL21 (DE3) transformed with pET-*cpxR*; Amp^r^	This study
DE3/pET-*esrB*	BL21 (DE3) transformed with pET-*esrB*; Amp^r^	This study
DE3/pET-*esrC*	BL21 (DE3) transformed with pET-*esrC*; Amp^r^	(49)
DE3/pET-*eseE*	BL21 (DE3) transformed with pET-*eseE*; Amp^r^	This study
Plasmids		
pWSK29	Low copy plasmid, promoterless, Amp^r^	(50)
pWSK29-*cpxR*_−762 to −1_ - *cpxA*-2HA	pWSK29 with *cpxR*_−762 to −1_-*cpxA*-2HA; Amp^r^	This study
pWSK29-*cpxA*_−528 to −1_ - *cpxA*-2HA	pWSK29 with *cpxA*_−528 to −1_-*cpxA*-2HA; Amp^r^	This study
pWSK29-*cpxA-*2HA	pWSK29 with *cpxA*-2HA; Amp^r^	This study
pWSK29-*cpxR*-2HA	pWSK29 with *cpxR*-2HA; Amp^r^	This study
pWSK29-*cpxR-cpxA*-2HA	pWSK29 with *cpxR-cpxA*-2HA; Amp^r^	This study
pWSK29-*esrB*-FLAG	pWSK29 with *esrB*_−610 to −1_-*esrB*-2HA; Amp^r^	This study
pWSK29-*esrC*-FLAG	pWSK29 with *esrC*_−420 to −1_-*esrC*-2HA; Amp^r^	(49)
pRE112	pGP704 suicide plasmid, *pir* dependent, Cm^r^, *oriT*, *oriV*, *sacB*	(51)
pET-21a	Prokaryotic expression plasmid; Amp^r^	Novagen
pJN105	*araC*-P_BAD_ cassette cloned in pBBR1MCS-5; Gm^r^	(37)
pJN105-*cpxR*-2HA	pJN105 with *cpxR*-2HA; Gm^r^	This study
pFPV25	Vector with promoterless *gfp* gene; Amp^r^	(52)
pFPV-*escC*_−200 to −1_ -*gfp*	−200 to −1 of *escC* gene inserted upstream of a promoterless *gfp* in pFPV25; Amp^r^	(4)
pFPV-*cpxR*_−226 to −1_ -*gfp*	−226 to −1 of *cpxR* gene inserted upstream of a promoterless *gfp* in pFPV25; Amp^r^	This study
pKD46	Red helper plasmid; Amp^r^	(53)
pSU315	Template plasmid with FRT recognition target site and 2 HA tag sequence; Amp^r^, Km^r^	(54)

^*a*^ Col, colistin; Km, kanamycin; Amp, ampicillin; Cm, chloramphenicol; Gm, gentamycin; r, resistance; s, sensitivity.

### Strain and plasmid construction

The *cpxA* gene was deleted from the chromosome of *E. piscicida* PPD130/91 by *sacB*-based allelic exchange, as previously described (28, 51). Similarly, the Δ*cpxR* strain and the Δ*cpxA*Δ*cpxR* strain were constructed. Using the λ Red recombination system (53), *tnaA* was deleted in *E. piscicida* PPD130/91 to obtain the Δ*tnaA* strain and the Δ*cpxA*Δ*tnaA* strain. To label the chromosomal copy of *cpxA* with DNA encoding the 2 HA epitope, the λ Red recombination system was used as previously described (54). To tag the chromosomal copy of *esrC* with the FLAG epitope, *sacB*-based allelic exchange was used as described previously (28, 51). The resulting colonies were probed with rabbit anti-HA and mouse anti-FLAG antibodies.

The primers used in this study are described in Table 2. The *cpxA* gene, together with the promoter upstream of *cpxR*, was amplified by overlapping PCR using primers pWSK-*cpxR*_−762 to −1_-*cpxA*-2HA-for, pWSK-*cpxR*_−762 to −1_-*cpxA*-2HA-int-rev, pWSK-*cpxR*_−762 to −1_-*cpxA*-2HA-int-for, and pWSK-*cpxA*-2HA-rev. The *cpxA* gene, together with the promoter upstream of *cpxA,* was amplified by pWSK29-*cpxA*_-528 to -1_-*cpxA*-2HA-for and pWSK29-*cpxA*-2HA-rev before being ligated into the XbaI and EcoRI restriction sites of pWSK29 (50). The sequence encoding the *cpxR* gene and its ribosome binding site was amplified using primers pJN-*cpxR*-2HA-for and pJN-*cpxR*-2HA-rev and subsequently inserted into the EcoRI and XbaI restriction sites of pJN105 (37) to produce the plasmid pJN-*cpxR*-2HA. The promoters of *escC*, *cpxA,* and *cpxR* in *E. piscicida* were amplified by PCR and then inserted into the EcoRI and XbaI restriction sites of pFPV25 (52). The full-length *cpxR*, *esrB*, and *esrC* genes were amplified using the primers listed in Table 2 and inserted into the BamHI and XhoI restriction sites of pET21a. All cloning operations were performed by using the ClonExpress II One Step Cloning Kit. The plasmids obtained were verified by DNA sequencing before being introduced into *E. piscicida* strains or *E. coli* BL21(DE3).

**TABLE 2:** Oligonucleotides used in this study

Primer	Nucleotide sequence (5′- 3′)
*cpxA*-for	GCTCTAGACGCTTCTGCAAGAACTTCTT
*cpxA*-int-rev	TACGATATTCTCGAACGCCCAAAAAATGGCAAAAATACG
*cpxA*-int-for	GCGTTCGAGAATATCGTACGCAAC
*cpxA*-rev	GCTCTAGAGTTATTATCGCAGTAGGGCT
*cpxR*-for	GCTCTAGAGCTTAATCAGCCGCCCAGTA
*cpxR*-int-rev	GAATTCAGTACCGGTGAGAGCGACGATAACCTCAAA
*cpxR*-int-for	CTCACCGGTACTGAATTCACGCTG
*cpxR*-rev	GCTCTAGATAGCGGGCCGGATCAGATAC
*tnaA*-red-for	CAAATTTATATTCCGGTGCTGATCAAGAAGCGTGAGCAAGAGAAGTGTGTAGGCTGGAGCTGCTTC
*tnaA*-red-rev	GATAATAAAGTCCATGTGCGTCTGGGTATAGGTGGCGCGCGGAATATATGAATATCCTCCTTA
pWSK29-*cpxR*_−762 to −1_- *cpxA*-2HA-for	CGCGGTGGCGGCCGCTCTAGAAGTTCACCCAAAGGTTGCTT
pWSK29-*cpxR*_−762 to −1_- *cpxA*-2HA-int-rev	TGGGTGTTACCTCCTGACGCAAAATACGATATCAAATGCCGCT
pWSK29-*cpxR*_−762 to −1_- *cpxA*-2HA-int-for	GCGTCAGGAGGTAACACCCAATGATCAATAGCCTGACGGC
pWSK29-*cpxA*_−528 to −1_- *cpxA*-2HA-for	CGCGGTGGCGGCCGCTCTAGACTTAAAGAGCTGCGTCAA
pWSK29-*cpxA*-2HA-for	CGCGGTGGCGGCCGCTCTAGAATGATCAATAGCCTGACG
pWSK29-*cpxA*-2HA-rev	GATAAGCTTGATATCGAATTCTTAAGCGTAATCTGGAACATCGTATGGGTAGGCTTTGCCCGTGCC
pWSK29-*cpxR*-2HA-for	GGTGGCGGCCGCTCTAGAAAAACCGTGGCGCGTAGTCT
pWSK29-*cpxR*-2HA-rev	GATAAGCTTGATATCGAATTCTTAAGCGTAATCTGGAACATCGTATGGGTATGAAACGGATACCATCA
pWSK29-*esrB*-flag-for	GGGCTGCAGGAATTCGATATCGGAGAGACCTCCCAATCG
pWSK29-*esrB*-flag-rev	CTATAGGGCGAATTGGGTACCTTATTTATCGTCGTCATCTTTGTAGTCAAACTCCAGAACCCCCA
pJN105-*cpxR*-2HA-for	GTTTTTTTGGGCTAGCACCCATACAGCTGAAAACAT
pJN105-*cpxR*-2HA-rev	CGCGGTGGCGGCCGCTCTAGATTAAGCGTAATCTGGAACATCGTATGGGTATGAAACGGATACCAT
pJN105-*cpxR*_D151A_-2HA- int-rev	AATGCTGGACGCCAGCATCGATCTGCTGCTACTG
pJN105-*cpxR*_D151A_-2HA- int-for	AGCATCGATCTGCTGCTACTGGCTGTCATGATGCCGAAGAAAAA
pFPV-*cpxR*_−226 to −1_-*gfp-*for	CCCTTTCGTCTTCAAGAATTCTCCCCTTCATCGGTGTATACGGCGA
pFPV-*cpxR*_−226 to −1_-*gfp-*rev	TGTATATCTCCTTCTTAAATCTAGATGGGTGTTACCTCCTGAC
pSU315-*cpxA*-2HA-for	ACGCTGTGGCTACCGCTCTCCACGCGCAGCGGCACGGGCAAAGCCTATCCGTATGATGTGCCGGACTATGCGTATCCGTATGATGTTCCTGAT
pSU315-*cpxA*-2HA-rev	AGGTTCGAAGAGTACGATATTCAGCATTTGCGCCCCCAGATGTCAGGGCATATGAATATCCTCCTTAG
*esrC*-FLAG-for	GAGAGCTCACCTGATTGCCGCGAACTCAG
*esrC*-FLAG-int-rev	CTTGTCATCGTCGTCCTTGTAATCGCCGGCGCGGTGGTGAAGGCTG
*esrC*-FLAG-int-for	GGACGACGATGACAAGTAAACACGGTAAGGAGCCTATAT
*esrC*-FLAG-rev	CTTCTAGAGACAGATTCACCAGCTTATTG
pET-*cpxR*-for	CAGCAAATGGGTCGCGGATCCATGAATAAGATATTACTGGT
pET-*cpxR*-rev	GTGGTGGTGGTGGTGCTCGAGTGAAACGGATACCATCA
pET-*esrB*-for	CAGCAAATGGGTCGCGGATCCATGACTATTTCTATTTTGCCTC
pET-*esrB*-rev	GTGGTGGTGGTGGTGCTCGAGAAACTCCAGAACCCCCAG
*cpxR-cpxA*-for	CATCGATCTGCTGCTACTGGATGTCATGATGCCGAAG
*cpxR-cpxA*-rev	GTCCTTCACTGGTCACCAGCAGCAGACGCTG
EMSA-*escC*_−200_-for	GCCCGAACACCCGAGAAAAACCAACGTA
EMSA-*escC*_−1_-rev	AGTGAAACCTCCTATTGACTACCGATGAGGTGATGCTAGAACGC
EMSA-*cpxR*_-226_-for	TCCCCTTCATCGGTGTATACGGCGA
EMSA-*cpxR*_+30_-rev	ATCGTCATCAACCAGTAATATCTTATTCATTGGG
*cpxR*-qfor	GATCAATAGCCTGACGGCGCGTATT
*cpxR*-qrev	ACCACAGCAGATCGTTGGGGGGGTC
*cpxA*-qfor	CGCCTCTTTCGCGCCATCG
*cpxA*-qrev	GCCACAGCAGCAGCGGTGA
*escC*-qfor	GCCAACACGCCATCCATCCC
*escC*-qrev	TGAATCGCTCAAGCCGCTGA
*eseB*-qfor	TGGATAAAGGGGAGCTCGAC
*eseB*-qrev	ATTTGTTCATCTCGGCCAGC
*escA*-qfor	CTATGCCTGCCAGCTCTTCG
*escA*-qrev	CCTGACCGAAGCAGAAGACG
*eseC*-qfor	AGGAGACACAGCCATGAACA
*eseC*-qrev	GAAACTCGCGCTTAGGATCG
*eseD*-qfor	ATCAACAGACGCAGCAAAGC
*eseD*-qrev	CATAGCCGACGCCGACAATC
*eseE*-qfor	GCCCGCAATGACGATGGACAGG
*eseE*-qrev	TCCGCCAGCATCACATCCGTCA
*rpoB*-qfor	TCAGATCCGCGGCGTAACCTA
*rpoB*-qrev	GCTGTCGAAGAATACGCCCGG
*gyrB*-qfor	TCGTCACCATTCACAGTGACAAC
*gyrB*-qrev	GTAGATTTGCTCATGGACATGGCC
5′-RACE-outer-for	GCTGATGGCGATGAATGAACACTG
5′-RACE-outer-rev	GGCGATCTCTTGCTGTAACGTGTGAAAGG
5′-RACE-inner-for	CGCGGATCCGAACACTGCGTTTGCTGGCTTTGATG
5′-RACE-inner-rev	TCGATCTGCTCGCCTAGCTTGCGCT

### Immunoblotting

*Edwardsiella piscicida* strains were subcultured at a 1:100 dilution into DMEM and grown statically at 25.0°C for 24 h. Extracellular proteins (ECPs) and bacterial lysates (total bacterial proteins, TBPs) were prepared as described by Liu et al. (4), and then similar amounts of proteins were loaded on the SDS-PAGE gels for immunoblotting. Phosphorylated proteins were isolated on Mn^2+^ Phos-Tag SDS-PAGE gel (12.0% acrylamide, 50.0 µM Phos-Tag [Wako, JPN], 100.0 µM MnCl_2_) prior to immunoblotting. Specifically, transfer buffer containing 1.0 mM EDTA (Ethylene diamine tetraacetic acid: Shenggong, China) was used to remove Mn^2+^ from the gel before proteins in the gels were transferred to the PVDF membranes (Millipore) and probed with rabbit anti-HA (1:3,000) (Sigma), mouse anti-FLAG (1:3,000) (Dia-an), rabbit anti-DnaK (1:2,000) (Invitrogen), rabbit anti-EseB (1:3,000) (55), rabbit anti-EseD (1:3,000) (56), rabbit anti-EseG (1:3,000) (57), rabbit anti-EseJ (1:3,000) (58), rabbit anti-CpxR (1:1,000), and mouse anti-RpoA (1:5,000) (Biolegend) antibodies and being incubated with horseradish peroxidase (HRP)-conjugated goat anti-rabbit (or mouse) IgG (1:2,000; Millipore, USA). Rabbit anti-CpxR antibody was produced by Genscript, China, by immunizing rabbits with keyhole limpet hemocyanin (KLH)-conjugated peptides of CpxR (CpxR aa 117–130; RSNWNEQQQNSDSG) and purified using the specific peptide as the ligand. Antigen-antibody complexes were detected using SuperSignal West Pico chemiluminescent substrate (Thermo) and imaged using a ChemiDoc MP imaging system (Bio-Rad, USA).

### Autoaggregation and biofilm formation assay

*Edwardsiella piscicida* strains were subcultured in glass tubes at a 1:100 dilution in DMEM. Each of the *E. piscicida* strains studied had similar growth curves. Their autoaggregation was imaged at 24 hours post-subculture (hps). Meanwhile, *E. piscicida* strains were subcultured at a 1:100 dilution in a 24-well tissue culture plate, horizontally embedded with coverslips and kept static. At 24 hps, the culture supernatants were carefully removed, and the coverslips were gently rinsed twice with pre-warmed PBS (phosphate-buffered saline) to remove the floating bacteria. The formed biofilm was fixed and stained with crystal violet as described by Gao et al. (3). The OD_630_ values of the biofilms solubilized with 1% SDS solution were measured using a spectrophotometer (BioTek Synergy Neo2 Hybrid, USA) to quantify the degree of biofilm formation.

### Immunofluorescence staining and confocal microscopy of *E. piscicida*

At 24 hps, *E. piscicida* strains attached to the coverslips were fixed in 4.0% PFA (paraformaldehyde), followed by immunofluorescence staining with mouse anti-EseB antibody (3) and donkey anti-mouse IgG antibody (Alexa 488; Molecular Probes), both at a 1:200 dilution. Images were captured using a confocal laser scanning microscope (Leica TCS SP8) with the objective of HC PL APO CS2 63 ×/1.40 oil fu, FLUO: gain (m): 680 offset (%): −0.17, TL-BF: gain (m): 331.6 Offset (%): 0.00. At 24 hps in DMEM, *E. piscicida* strains expressing green fluorescent protein (GFP) were imaged using a confocal microscope (Leica DMi8) with the objective 100 × oil fu/visual 200 ×, FLUO: exposure (ms): 500, TL-BF exposure (ms): 20. The intensity of the GFP signal was measured using a multifunctional microplate detector (BioTek Synergy Neo2 Hybrid) with an excitation wavelength of 485 and an emission wavelength of 528.

### RNA isolation, cDNA synthesis, and quantitative real-time PCR

*E. piscicida* strains were subcultured in DMEM at a 1:100 dilution for 24 h prior to measuring bacterial OD_540_ nm. Cell densities were normalized, and an equivalent amount of bacteria per strain was used for RNA isolation using the RNeasy Mini Kit (Qiagen), followed by Dnase I treatment to remove genomic DNA contamination. First-strand cDNA was synthesized using PrimeScript Reverse Transcriptase (Thermo Scientific, Wilmington, USA). Real-time PCR was performed on a CFX96 real-time system (Bio-Rad) using SYBR green master mix (Bio-Rad). Both *rpoB* and *gyrB* were used as the reference genes to calculate and determine the relative expression levels of the target genes as described by Vandesompele et al. (59). The qRT-PCR products were electrophoresed and photographed using a Gel Image Analyzing JS-1800 system (Peiqing, CHN).

### 5′-RACE assay

The First Choice RLM-RACE Kit (Thermo) was used for 5′-RACE according to the manufacturer’s instructions with some modifications. Briefly, total RNA (1.6 µg) isolated from the WT strain was ligated with 1.0 µL 5′ RACE adapter (0.3 µg/µL) in a mixture (10.0 µL) containing 1 × T4 RNA ligase buffer and 2.0 µL T4 RNA ligase (2.5 U/µL) and incubated at 37°C for 1 h. Total adapted RNA was then treated with DNase I to remove genomic DNA contamination and reverse transcribed into cDNA using the Revert Aid First Strand cDNA Synthesis Kit (Thermo). Using the first-strand cDNA as a template, target DNA fragments were amplified by PCR using the outer and inner pair of 5′-RACE primers and cloned into the T vector for sequencing. The adapter sequence was used to determine the transcription start sites of the target gene.

### Protein expression and purification, and electrophoretic mobility shift assay (EMSA)

His_6_-tagged CpxR, EseE, and EsrB were each overexpressed in *E. coli* BL21(DE3) using pET21a as a vector. For purification, the recombinant *E. coli* BL21(DE3) strain was cultured overnight and then subcultured at 1:100 in fresh LB broth, and cultured at 37.0°C at 200.0 rpm until OD_600_ reached 0.5. Then, IPTG (isopropyl-beta-D-thiogalactopyranoside) (Shenggong, China) was added at a final concentration of 0.2 mM, and protein expression was induced overnight at 16.0°C at 120.0 rpm. Bacteria were pelleted at 12,000 *× g* for 5.0 min at 4.0°C before being resuspended in PBS and sonicated in an ice bath for 5.0 min (power 15.0 W, ultrasound 2.0 s, interval 3.0 s). The supernatants from the cell lysates were obtained by centrifugation at 13,000 × *g* at 4.0°C for 20.0 minutes. The supernatants were filtered through a 0.22 µm ultrafiltration membrane (Millipore) and then subjected to Ni^+^ affinity chromatography for protein purification as described in the Qiagen manual. Full-length EsrC-His_6_ was induced in *E. coli* BL21(DE3), which was transformed with pET21a-*esrC* (49), and the soluble EsrC-His_6_ protein in the bacterial lysate was used for the EMSA.

DNA probes were prepared by PCR using the primer pairs EMSA-*escC*_−200_*-*for/EMSA-*escC*_-1_-rev, EMSA-*cpxR*_-226_*-*for/EMSA-*cpxR*-rev, and EMSA-*cpxR*_+2 to +160_*-*for/EMSA-*cpxR*_+2 to +160_ rev (Table 2). His_6_-tagged CpxR, EseE, EsrB, and EsrC at different concentrations were mixed with the DNA fragments labeled at the 5′-end with Cy3 (Shenggong, China) in a 20.0 µL EMSA reaction system containing 20.0 mM Tris-HCl (pH 7.5), 50.0 mM MgCl_2_, and 5.0% (vol/vol) glycerol for 2 h at 25.0°C before loading on a 5.0% native polyacrylamide gel. His_6_-tagged CpxR was incubated with or without 0.4 M lithium potassium acetyl phosphate (AcP) (Sigma) as the phosphate donor in the kinase buffer at 30.0°C for 1 h, then the protein mixtures were added to the reaction system for EMSA. Electrophoresis was performed using 0.5 × Tris-borate-EDTA buffer.

### Statistical analysis

All data were analyzed using the one-way ANOVA in the Statistical Package for the Social Sciences (SPSS) and expressed as the mean ± standard error of the mean (SEM) or as the mean ± standard deviation (SD). *P* values less than 0.05 were considered statistically significant.

## References

[1] Mowbray EE, Buck G, Humbaugh KE, Marshall GS. 2003. Maternal colonization and neonatal sepsis caused by Edwardsiella tarda. Pediatrics 111:e296–e298. doi:10.1542/peds.111.3.e296 12612287

[2] Leung KY, Siame BA, Tenkink BJ, Noort RJ, Mok YK. 2012. Edwardsiella tarda - virulence mechanisms of an emerging gastroenteritis pathogen. Microbes Infect 14:26–34. doi:10.1016/j.micinf.2011.08.005 21924375

[3] Gao ZP, Nie P, Lu JF, Liu LY, Xiao TY, Liu W, Liu JS, Xie HX. 2015. Type III secretion system translocon component EseB forms filaments on and mediates autoaggregation of and biofilm formation by Edwardsiella tarda. Appl Environ Microbiol 81:6078–6087. doi:10.1128/AEM.01254-15 26116669 PMC4551241

[4] Liu YL, He TT, Liu LY, Yi J, Nie P, Yu HB, Xie HX. 2019. The Edwardsiella piscicida type III translocon protein EseC inhibits biofilm formation by sequestering EseE. Appl Environ Microbiol 85:e02133-18. doi:10.1128/AEM.02133-18 30770403 PMC6450016

[5] Tan YP, Zheng J, Tung SL, Rosenshine I, Leung KY. 2005. Role of type III secretion in Edwardsiella tarda virulence. Microbiology (Reading) 151:2301–2313. doi:10.1099/mic.0.28005-0 16000720

[6] Zheng J, Li N, Tan YP, Sivaraman J, Mok YK, Mo ZL, Leung KY. 2007. EscC is a chaperone for the Edwardsiella tarda type III secretion system putative translocon components EseB and EseD. Microbiology (Reading) 153:1953–1962. doi:10.1099/mic.0.2006/004952-0 17526852

[7] Yi J, Xiao SB, Zeng ZX, Lu JF, Liu LY, Laghari ZA, Nie P, Yu HB, Xie HX. 2016. EseE of Edwardsiella tarda augments secretion of translocon protein EseC and expression of the escC~eseE operon. Infect Immun 84:2336–2344. doi:10.1128/IAI.00106-16 27271743 PMC4962638

[8] Kostakioti M, Hadjifrangiskou M, Hultgren SJ. 2013. Bacterial biofilms: development, dispersal, and therapeutic strategies in the dawn of the postantibiotic era. Cold Spring Harb Perspect Med 3:a010306. doi:10.1101/cshperspect.a010306 23545571 PMC3683961

[9] Rumbaugh KP, Sauer K. 2020. Biofilm dispersion. Nat Rev Microbiol 18:571–586. doi:10.1038/s41579-020-0385-0 32533131 PMC8564779

[10] Koo H, Allan RN, Howlin RP, Stoodley P, Hall-Stoodley L. 2017. Targeting microbial biofilms: current and prospective therapeutic strategies. Nat Rev Microbiol 15:740–755. doi:10.1038/nrmicro.2017.99 28944770 PMC5685531

[11] Schilcher K, Horswill AR. 2020. Staphylococcal biofilm development: structure, regulation, and treatment strategies. Microbiol Mol Biol Rev 84:e00026-19. doi:10.1128/MMBR.00026-19 32792334 PMC7430342

[12] Chen HH, Chang CC, Yuan YH, Liaw SJ. 2020. A CpxR-regulated zapD gene involved in biofilm formation of uropathogenic Proteus mirabilis. Infect Immun 88:e00207-20. doi:10.1128/IAI.00207-20 32284373 PMC7309609

[13] Fadel F, Bassim V, Francis VI, Porter SL, Botzanowski T, Legrand P, Perez MM, Bourne Y, Cianférani S, Vincent F. 2022. Insights into the atypical autokinase activity of the Pseudomonas aeruginosa GacS histidine kinase and its interaction with RetS. Structure 30:1285–1297. doi:10.1016/j.str.2022.06.002 35767996

[14] Taylor PK, Zhang L, Mah TF. 2019. Loss of the two-component system TctD-TctE in Pseudomonas aeruginosa affects biofilm formation and aminoglycoside susceptibility in response to citric acid. mSphere 4:e00102-19. doi:10.1128/mSphere.00102-19 30842268 PMC6403454

[15] Alwis PA, Treerat P, Gong L, Deveson Lucas D, Allwood EM, Prescott M, Devenish RJ, Adler B, Boyce JD. 2020. Disruption of the Burkholderia pseudomallei two-component signal transduction system BbeR-BbeS leads to increased extracellular DNA secretion and altered biofilm formation. Vet Microbiol 242:108603. doi:10.1016/j.vetmic.2020.108603 32122607

[16] Shetty D, Abrahante JE, Chekabab SM, Wu X, Korber DR, Vidovic S. 2019. Role of CpxR in biofilm development: expression of key fimbrial, O-antigen and virulence operons of Salmonella Enteritidis. Int J Mol Sci 20:5146. doi:10.3390/ijms20205146 31627387 PMC6829429

[17] Matter LB, Ares MA, Abundes-Gallegos J, Cedillo ML, Yáñez JA, Martínez-Laguna Y, De la Cruz MA, Girón JA. 2018. The CpxRA stress response system regulates virulence features of avian pathogenic Escherichia coli. Environ Microbiol 20:3363–3377. doi:10.1111/1462-2920.14368 30062827

[18] Gahlot DK, Wai SN, Erickson DL, Francis MS. 2022. Cpx-signalling facilitates Hms-dependent biofilm formation by Yersinia pseudotuberculosis. NPJ Biofilms Microbiomes 8:13. doi:10.1038/s41522-022-00281-4 35351893 PMC8964730

[19] Lv Y, Xiao J, Liu Q, Wu H, Zhang Y, Wang Q. 2012. Systematic mutation analysis of two-component signal transduction systems reveals EsrA-EsrB and PhoP-PhoQ as the major virulence regulators in Edwardsiella tarda. Vet Microbiol 157:190–199. doi:10.1016/j.vetmic.2011.12.018 22227416

[20] Raivio TL. 2014. Everything old is new again: an update on current research on the Cpx envelope stress response. Biochim Biophys Acta 1843:1529–1541. doi:10.1016/j.bbamcr.2013.10.018 24184210

[21] Wolfe AJ, Parikh N, Lima BP, Zemaitaitis B. 2008. Signal integration by the two-component signal transduction response regulator CpxR. J Bacteriol 190:2314–2322. doi:10.1128/JB.01906-07 18223085 PMC2293188

[22] Hunke S, Keller R, Müller VS. 2012. Signal integration by the Cpx-envelope stress system. FEMS Microbiol Lett 326:12–22. doi:10.1111/j.1574-6968.2011.02436.x 22092888

[23] Vogt SL, Raivio TL. 2012. Just scratching the surface: an expanding view of the Cpx envelope stress response. FEMS Microbiol Lett 326:2–11. doi:10.1111/j.1574-6968.2011.02406.x 22092948

[24] Guest RL, Raivio TL. 2016. Role of the Gram-negative envelope stress response in the presence of antimicrobial agents. Trends Microbiol 24:377–390. doi:10.1016/j.tim.2016.03.001 27068053

[25] Liu J, Thanikkal EJ, Obi IR, Francis MS. 2012. Elevated CpxR∼P levels repress the Ysc–Yop type III secretion system of Yersinia pseudotuberculosis. Res Microbiol 163:518–530. doi:10.1016/j.resmic.2012.07.010 22842077

[26] De la Cruz MA, Pérez-Morales D, Palacios IJ, Fernández-Mora M, Calva E, Bustamante VH. 2015. The two-component system CpxR/A represses the expression of Salmonella virulence genes by affecting the stability of the transcriptional regulator HilD. Front Microbiol 6:807. doi:10.3389/fmicb.2015.00807 26300871 PMC4526804

[27] León-Montes N, Nava-Galeana J, Rodríguez-Valverde D, Soria-Bustos J, Rosales-Reyes R, Rivera-Gutiérrez S, Hirakawa H, Ares MA, Bustamante VH, De la Cruz MA. 2022. The two-component system CpxRA represses Salmonella Pathogenicity Island 2 by directly acting on the ssrAB regulatory operon. Microbiol Spectr 10:e0271022. doi:10.1128/spectrum.02710-22 36073960 PMC9603713

[28] Zheng J, Tung SL, Leung KY. 2005. Regulation of a type III and a putative secretion system in Edwardsiella tarda by EsrC is under the control of a two-component system, EsrA-EsrB. Infect Immun 73:4127–4137. doi:10.1128/IAI.73.7.4127-4137.2005 15972502 PMC1168592

[29] Shao S, Li C, Zhao L, Zhang Y, Yin K, Wang Q. 2021. Interplay between ferric uptake regulator Fur and horizontally acquired virulence regulator EsrB coordinates virulence gene expression in Edwardsiella piscicida. Microbiol Res 253:126892. doi:10.1016/j.micres.2021.126892 34673373

[30] Ahmed MAH, Cai J, Zhang Y, Yin K, Wang Q, Shao S. 2022. The cross-regulation between two-component system BasS-BasR and ferric uptake regulator Fur in virulence gene expression in Edwardsiella piscicida. Aquaculture 559:738405. doi:10.1016/j.aquaculture.2022.738405

[31] Kumar A, Sperandio V. 2019. Indole signaling at the host-microbiota-pathogen interface. MBio 10:e01031-19. doi:10.1128/mBio.01031-19 31164470 PMC6550529

[32] Lee JH, Lee J. 2010. Indole as an intercellular signal in microbial communities. FEMS Microbiol Rev 34:426–444. doi:10.1111/j.1574-6976.2009.00204.x 20070374

[33] Cui B, Chen X, Guo Q, Song S, Wang M, Liu J, Deng Y. 2022. The cell-cell communication signal indole controls the physiology and interspecies communication of Acinetobacter baumannii. Microbiol Spectr 10:e0102722. doi:10.1128/spectrum.01027-22 35862954 PMC9431217

[34] Han Y, Yang C-L, Yang Q, Qi Z, Liu W, Xu Z-H, Zhu W-M, Bossier P, Zhang X-H. 2011. Mutation of tryptophanase gene tnaA in Edwardsiella tarda reduces lipopolysaccharide production, antibiotic resistance and virulence. Environ Microbiol Rep 3:603–612. doi:10.1111/j.1758-2229.2011.00269.x 23761341

[35] McShan AC, Anbanandam A, Patnaik S, De Guzman RN. 2016. Characterization of the binding of hydroxyindole, indoleacetic acid, and morpholinoaniline to the Salmonella type III secretion system proteins SipD and SipB. ChemMedChem 11:963–971. doi:10.1002/cmdc.201600065 26990667 PMC5010876

[36] Cho THS, Wang J, Raivio TL. 2023. NlpE is an OmpA-associated outer membrane sensor of the Cpx envelope stress response. J Bacteriol 205:e0040722. doi:10.1128/jb.00407-22 37022159 PMC10127795

[37] Khlebnikov A, Skaug T, Keasling JD. 2002. Modulation of gene expression from the arabinose-inducible araBAD promoter. J Ind Microbiol Biotechnol 29:34–37. doi:10.1038/sj.jim.7000259 12080425

[38] Pogliano J, Lynch AS, Belin D, Lin EC, Beckwith J. 1997. Regulation of Escherichia coli cell envelope proteins involved in protein folding and degradation by the Cpx two-component system. Genes Dev 11:1169–1182. doi:10.1101/gad.11.9.1169 9159398

[39] Liu J, Obi IR, Thanikkal EJ, Kieselbach T, Francis MS. 2011. Phosphorylated CpxR restricts production of the RovA global regulator in Yersinia pseudotuberculosis. PLoS One 6:e23314. doi:10.1371/journal.pone.0023314 21876746 PMC3158067

[40] Guan Y, Yin K, Zhou M, Yang M, Zhang Y, Liu X, Wang Q. 2018. EsrB negatively regulates expression of the glutamine sythetase GlnA in the fish pathogen Edwardsiella piscicida. FEMS Microbiol Lett 365:fny007. doi:10.1093/femsle/fny007 29346648

[41] Flemming HC, Wingender J, Szewzyk U, Steinberg P, Rice SA, Kjelleberg S. 2016. Biofilms: an emergent form of bacterial life. Nat Rev Microbiol 14:563–575. doi:10.1038/nrmicro.2016.94 27510863

[42] Shimizu T, Ichimura K, Noda M. 2016. The surface sensor NlpE of enterohemorrhagic Escherichia coli contributes to regulation of the type III secretion system and flagella by the Cpx response to adhesion. Infect Immun 84:537–549. doi:10.1128/IAI.00881-15 26644384 PMC4730559

[43] Moreira CG, Palmer K, Whiteley M, Sircili MP, Trabulsi LR, Castro AFP, Sperandio V. 2006. Bundle-forming pili and EspA are involved in biofilm formation by enteropathogenic Escherichia coli. J Bacteriol 188:3952–3961. doi:10.1128/JB.00177-06 16707687 PMC1482920

[44] Tierney AR, Rather PN. 2019. Roles of two-component regulatory systems in antibiotic resistance. Future Microbiol 14:533–552. doi:10.2217/fmb-2019-0002 31066586 PMC6526388

[45] Bansal T, Englert D, Lee J, Hegde M, Wood TK, Jayaraman A. 2007. Differential effects of epinephrine, norepinephrine, and indole on Escherichia coli O157:H7 chemotaxis, colonization, and gene expression. Infect Immun 75:4597–4607. doi:10.1128/IAI.00630-07 17591798 PMC1951185

[46] Kohli N, Crisp Z, Riordan R, Li M, Alaniz RC, Jayaraman A. 2018. The microbiota metabolite indole inhibits Salmonella virulence: involvement of the PhoPQ two-component system. PLoS One 13:e0190613. doi:10.1371/journal.pone.0190613 29342189 PMC5771565

[47] Chakraborty S, Li M, Chatterjee C, Sivaraman J, Leung KY, Mok YK. 2010. Temperature and Mg^2+^ sensing by a novel PhoP-PhoQ two-component system for regulation of virulence in Edwardsiella tarda. J Biol Chem 285:38876–38888. doi:10.1074/jbc.M110.179150 20937832 PMC2998078

[48] Ling SHM, Wang XH, Xie L, Lim TM, Leung KY. 2000. Use of green fluorescent protein (GFP) to study the invasion pathways of Edwardsiella tarda in in vivo and in vitro fish models. Microbiology (Reading) 146 (Pt 1):7–19. doi:10.1099/00221287-146-1-7 10658647

[49] Sun SS, He TT, Zhang SY, Yu X-J, Chen C, Laghari ZA, Nie P, Xie HX. 2024. T3SS protein EsrC binds to the lacI-like operator of type 1 fimbrial operon to suppress adhesion of Edwardsiella piscicida. Appl Environ Microbiol 90:e0086224. doi:10.1128/aem.00862-24 39058035 PMC11337838

[50] Wang RF, Kushner SR. 1991. Construction of versatile low-copy-number vectors for cloning, sequencing and gene expression in Escherichia coli. Gene 100:195–199. 2055470

[51] Edwards RA, Keller LH, Schifferli DM. 1998. Improved allelic exchange vectors and their use to analyze 987P fimbria gene expression. Gene 207:149–157. doi:10.1016/S0378-1119(97)00619-7 9511756

[52] Valdivia RH, Falkow S. 1996. Bacterial genetics by flow cytometry: rapid isolation of Salmonella typhimurium acid-inducible promoters by differential fluorescence induction. Mol Microbiol 22:367–378. doi:10.1046/j.1365-2958.1996.00120.x 8930920

[53] Datsenko KA, Wanner BL. 2000. One-step inactivation of chromosomal genes in Escherichia coli K-12 using PCR products. Proc Natl Acad Sci USA 97:6640–6645. doi:10.1073/pnas.120163297 10829079 PMC18686

[54] Uzzau S, Figueroa-Bossi N, Rubino S, Bossi L. 2001. Epitope tagging of chromosomal genes in Salmonella. Proc Natl Acad Sci USA 98:15264–15269. doi:10.1073/pnas.261348198 11742086 PMC65018

[55] Zhou Y, Liu LY, He TT, Laghari ZA, Nie P, Gao Q, Xie HX. 2016. Edwardsiella tarda EsaE (Orf19 protein) is required for the secretion of type III substrates, and pathogenesis in fish. Vet Microbiol 190:12–18. doi:10.1016/j.vetmic.2016.05.003 27283851

[56] Lu JF, Wang WN, Wang GL, Zhang H, Zhou Y, Gao ZP, Nie P, Xie HX. 2016. Edwardsiella tarda EscE (Orf13 protein) is a type III secretion system-secreted protein that is required for the injection of effectors, secretion of translocators, and pathogenesis in fish. Infect Immun 84:2–10. doi:10.1128/IAI.00986-15 26459509 PMC4693999

[57] Xie HX, Yu HB, Zheng J, Nie P, Foster LJ, Mok YK, Finlay BB, Leung KY. 2010. EseG, an effector of the type III secretion system of Edwardsiella tarda, triggers microtubule destabilization. Infect Immun 78:5011–5021. doi:10.1128/IAI.00152-10 20855515 PMC2981322

[58] Xie HX, Lu JF, Zhou Y, Yi J, Yu XJ, Leung KY, Nie P. 2015. Identification and functional characterization of the novel Edwardsiella tarda effector EseJ. Infect Immun 83:1650–1660. doi:10.1128/IAI.02566-14 25667268 PMC4363441

[59] Vandesompele J, De Preter K, Pattyn F, Poppe B, Van Roy N, De Paepe A, Speleman F. 2002. Accurate normalization of real-time quantitative RT-PCR data by geometric averaging of multiple internal control genes. Genome Biol 3:1–12. doi:10.1186/gb-2002-3-7-research0034 PMC126239 12184808

